# Supplementary material for: DEVOLUTION—A method for phylogenetic reconstruction of aneuploid cancers based on multiregional genotyping data
Source: Commun Biol. 2021 Sep 20;4:1103. doi: 10.1038/s42003-021-02637-6 (PMC8452746; doi:10.1038/s42003-021-02637-6)
Supplement: Supplementary file 2 — Supplementary information. [file 42003_2021_2637_MOESM2_ESM.pdf]

Supplementary information

S. Table 1 - Comparison between methods

S. Table 2-3 - Example of input data file

S. Figure 1 - Finding recurrent aberrations across biopsies

S. Figure 2-7 - Phylogenetic trees for all the neuroblastoma, Wilms tumor and Rhabdomyosarcoma cases included in the study.

S. Figure 8 - Contradictions resulting from parallel evolution or back mutations

S. Figure 9 - Contradictions in a complex dataset

S. Figure 10-12 - Comparison between phylogenetic trees based on sequencing data alone, SNP-array data alone and when they are used in unison for NB, WT and RMS

S. Figure 13 - Violin plots for the comparison between phylogenetic trees based on sequencing data alone, SNP-array data alone and when they are used in unison

S. Figure 14-17 - TRACERx analysis using DEVOLUTION and MAGOS

S. Figure 18 - Assessing alternative solutions

S. Figure 19-29 - Flow charts of the algorithm workflow

S. Figure 30 - Visualizing the MCF for each genetic alteration across the biopsies

S. Figure 31 - Incorporating user-controlled rules for avoiding imposition of illicit biological trajectories

S. Methods including S. Figure 32-41 - Detailed description of the algorithm

Supplementary Table 1

	Pyclone	SciClone	PhyloWGS	TITAN THetA	CloneSeeker	CHAT	DEVOLUTION
Multiple samples	Y	Y	Y*	N	N	N	Y
Sequence data	Required	Required	Required**	Required*	Optional	Optional	Optional
SNP array	Optional	Optional	No	No	Optional	Optional	Optional
Takes CNAs into account	Y*	Y*	Y	Y	Y	Y	Y
Allows subclonal CNA	N	N	Y	Y	Y	Y	Y
Resolves ovelapping CNAs with different endpoints	N	N	N	N	N	N	Y
Integrates information from multiple samples to infer the evolutionary relationship between events.	N	N	N	N	N	N	Y
Constructs a phylogeny	N	N	Y	N	N	N	Y
Can construct phylogenies based on CNAs alone.	N	N	N	N	N	N	Y
Comment	*Assumes that, when a CNA and SNA overlap, the point mutation resides in a region with homogeneous aneuploidy. Hence no subclonal CNA events are allowed.	*Focuses exclusively on SNV in copy-number neutral, loss of heteroztgosity (LOH)-free portions of the genome.	*Do not compare information between samples during the inference procedure. **Limited to WGS data.	*Limited to WGS data.	- Can read SNP-array data directly and/or sequence data to compute the number of clones, although no more than five and is limited to one single sample. - Does not infer the order of events.	- Does not integrate information from multiple samples. - Uses SNP-array data or sequencing data to infer the "CCF" (cancer cell fraction=cellular prevalence of SNVs in this paper). They do consider the order of the SNV relative to the CNA.	- Input data: MCFs inferred from SNP-array, WGS, WES and/or TDS in unison or separately. - There is no limit for the number of samples that can be analysed. - SNP-array data can be analysed alone. - Information about point mutations can be added to the segment file to be integrated in the process. - Subclones are inferred and their distribution across biopsies visualized in a phylogenetic tree.

Supplementary Table 1 Comparison between DEVOLUTION and available tools.

Supplementary Table 2

Tumor ID	Sample ID	Chr	Start	End	Med LogR	VAF	Type	Method	Cytoband/ Gene	Clone size (%)
WT13	ALL	2	209010574	209010574	NA	NA	p.R59Q	TDS	CRYGB	100
WT13	ALL	5	31421465	31421465	NA	NA	p.E1110K	TDS	DROSHA	100
WT13	ALL	11	192764	4238769	NA	NA	CNNI	SNP array	11p15p15	100
WT13	B1	2	216214492	216489509	-0,31	NA	LOSS	SNP array	2q35q35	40
WT13	B1	6	0	170919481	0,22	NA	GAIN	SNP array	WHOLE	40
WT13	B1	8	0	146267159	0,22	NA	GAIN	SNP array	WHOLE	40
WT13	B2	2	216214492	216489509	-0,73	NA	LOSS	SNP array	2q35q35	90
WT13	B2	6	204909	170913051	0,37	NA	GAIN	SNP array	WHOLE	90
WT13	B2	8	172417	146292734	0,37	NA	GAIN	SNP array	WHOLE	90
WT13	B3	2	216214492	216489509	-0,33	NA	LOSS	SNP array	2q35q35	60
WT13	B3	6	204909	170913051	0,22	NA	GAIN	SNP array	WHOLE	60
WT13	B3	8	172417	146292734	0,20	NA	GAIN	SNP array	WHOLE	60
WT13	B3	3	63411	197852564	0,19	NA	GAIN	SNP array	WHOLE	50
WT13	B3	12	189400	133818115	0,19	NA	GAIN	SNP array	WHOLE	50
WT13	B3	18	12842	78007784	0,17	NA	GAIN	SNP array	WHOLE	50
WT13	P	2	216214492	216489509	-0,72	NA	LOSS	SNP array	2q35q35	100
WT13	P	6	0	170919481	0,50	NA	GAIN	SNP array	WHOLE	100
WT13	P	8	0	146267159	0,50	NA	GAIN	SNP array	WHOLE	100
WT13	P	1	46650978	46650978	NA	0,4045	p.E226K	TDS	TSPAN1	100
WT13	P	2	16082317	16082317	NA	0,443386	p.P44L	TDS	MYCN	100
WT13	P	16	2347399	2347399	NA	0,387581	p.R732C	TDS	ABCA3	100
WT13	P	3	48623783	48623783	NA	0,247	p.R1178C	TDS	COL7A1	60

Supplementary Table 2 Example of input data file. A segment of the data obtained from SNP array analysis and parallel targeted deep sequencing (TDS). In the first column the tumor ID is indicated, in this case Wilms Tumor number 13. In the second column the biopsy name can be seen, indicating which aberrations have been found in which biopsy. Columns 3-5 shows the location of the alterations on the chromosomes. Columns 6 and 7 list the log2 median values for copy number aberrations av variant allele frequencies (VAF) for point mutations, respectively. Columns 8 and 10 indicates the type of aberration. A GAIN means that there is one extra copy of this particular gene segment. Similarly, a LOSS indicates that one copy of the gene segment has been lost. The rightmost column gives the fraction of the cells in that particular biopsy that harbor this aberration, denoted the mutated clone fraction (MCF).

Supplementary Figure 1

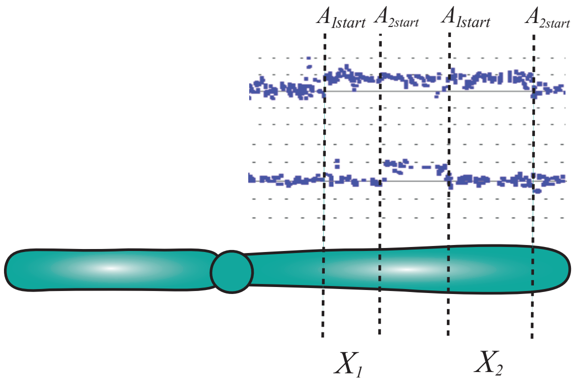

$$X_1 = \left\| A_{1start} - A_{2start} \right\| \leq co_{ev}$$

$$X_2 = \left\| A_{1end} - A_{2end} \right\| \leq co_{ev}$$

Supplementary Figure 1 Finding recurrent aberrations across biopsies. Schematic representation of the comparison the algorithm makes. To be categorized as the same aberration, alteration 1 and 2 must be localized on the same chromosome, harbor the same type of alteration (for example GAIN, LOSS, CNNI etc.) and should not belong to the stem. Alterations belonging to the stem are always considered as separate events. The difference between the alterations' edges must be smaller than or equal to the cutoff chosen by the user reflecting the uncertainty in the measurement of the events.

Supplementary Figure 2

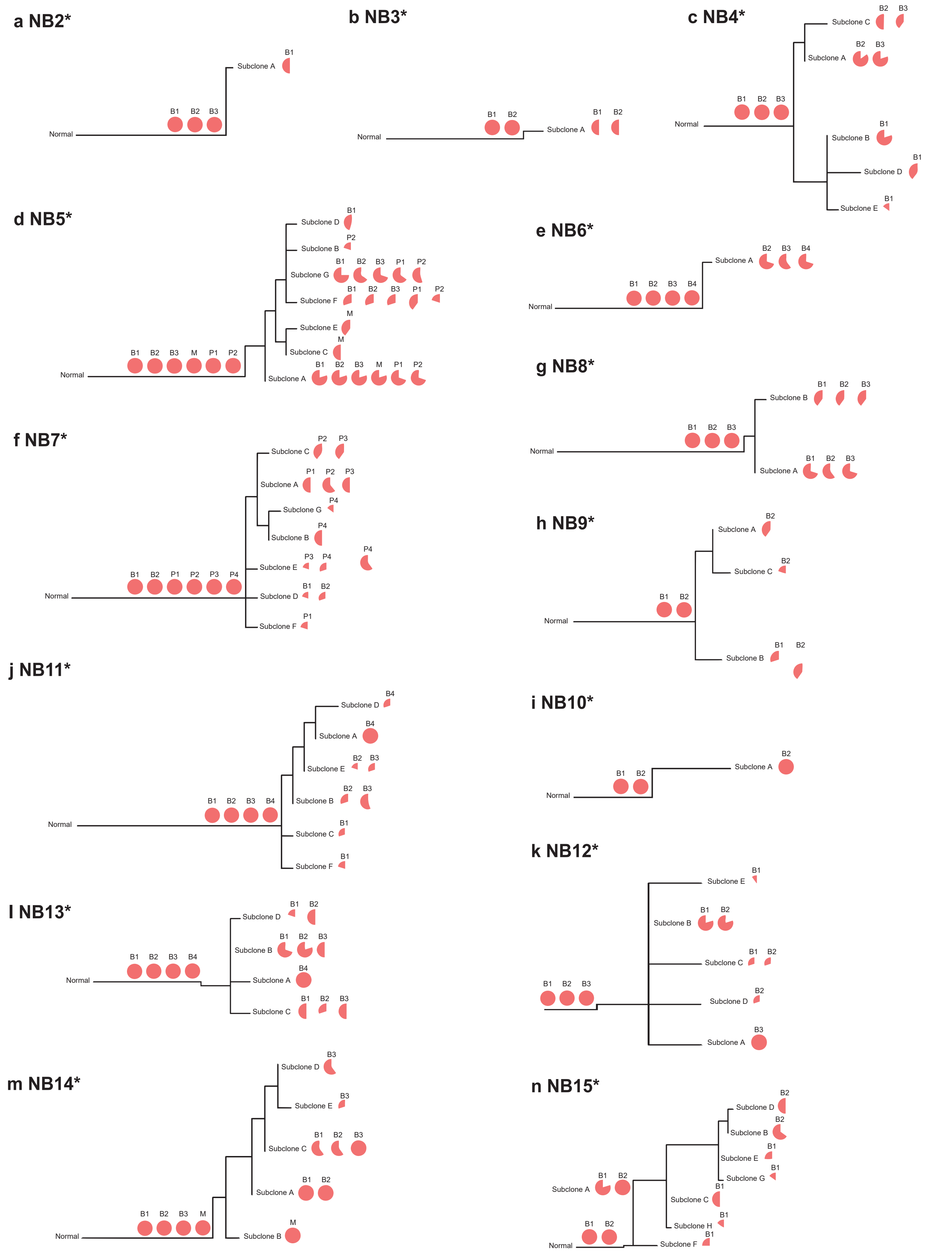

Supplementary Figure 2 Phylogenetic trees for NB2-15. a-n) At the stem, the available biopsies from each patient are visualized by filled pies. An asterix after the patient name indicates that the MP- and ML-trees for this tumor are identical. When they are not identical, both are displayed. The endpoints of the trees represent cell populations harboring distinct genomic profiles (subclones), whose fractions across samples are visualized as pie charts. Biopsies are available from the primary tumor before treatment (B) and after treatment (P), relapses (R), distant metastases (M), lymph node metastases (LN) and lung metastases (LU). The segment files used to produce the phylogenies can be found in Supplementary data 1 and the corresponding event matrices produced by DEVOLUTION in Supplementary data 2.

Supplementary Figure 3

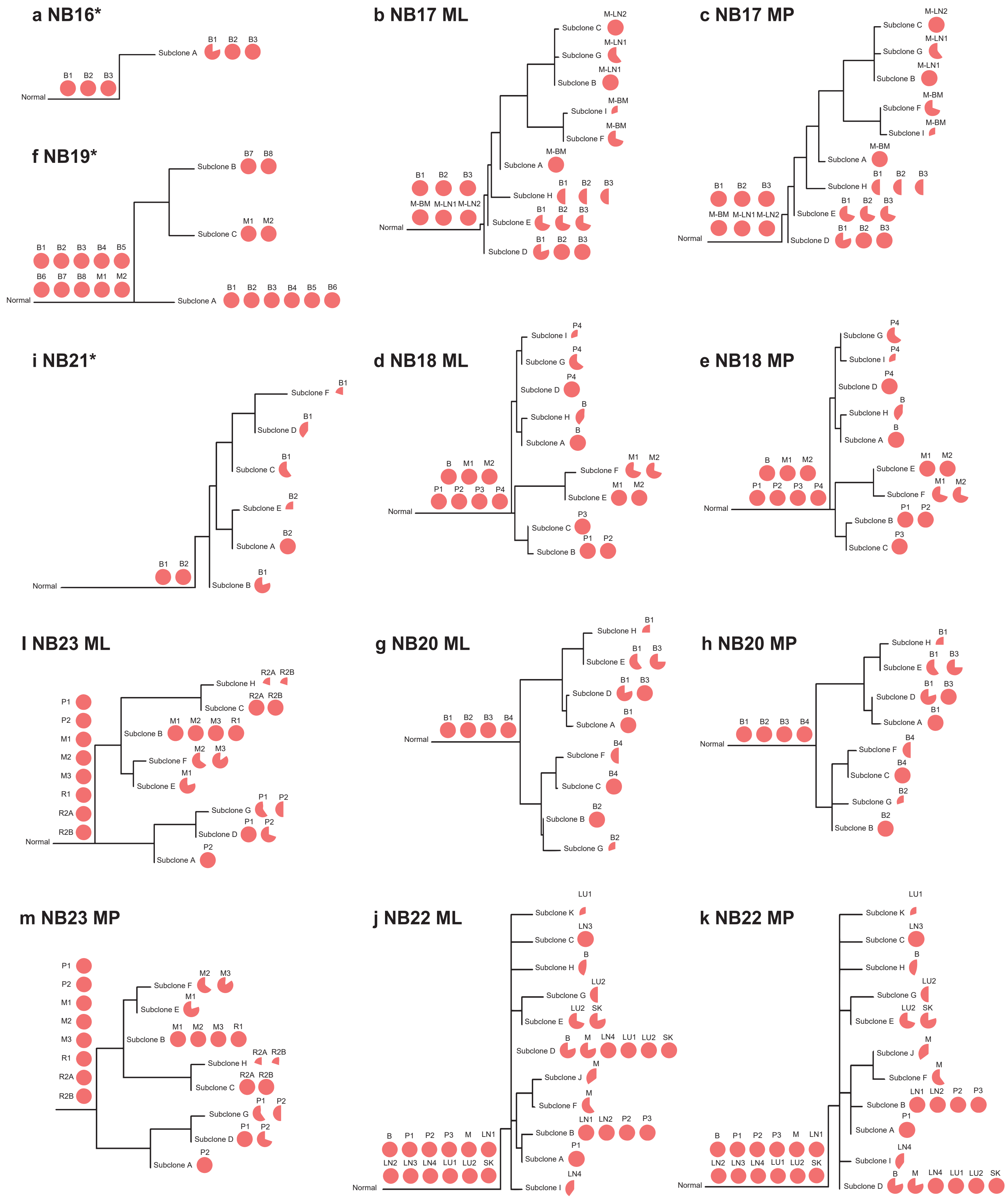

Supplementary Figure 3 Phylogenetic trees for NB16-23. a-m) At the stem, the available biopsies from each patient are visualized by filled pies. An asterix after the patient name indicates that the MP- and ML-trees for this tumor are identical. When they are not identical, both are displayed. The endpoints of the trees represent cell populations harboring distinct genomic profiles (subclones), whose fractions across samples are visualized as pie charts. Biopsies are available from the primary tumor before treatment (B) and after treatment (P), relapses (R), distant metastases (M), lymph node metastases (LN) and lung metastases (LU). The segment files used to produce the phylogenies can be found in Supplementary data 1 and the corresponding event matrices produced by DEVOLUTION in Supplementary data 2.

Supplementary Figure 4

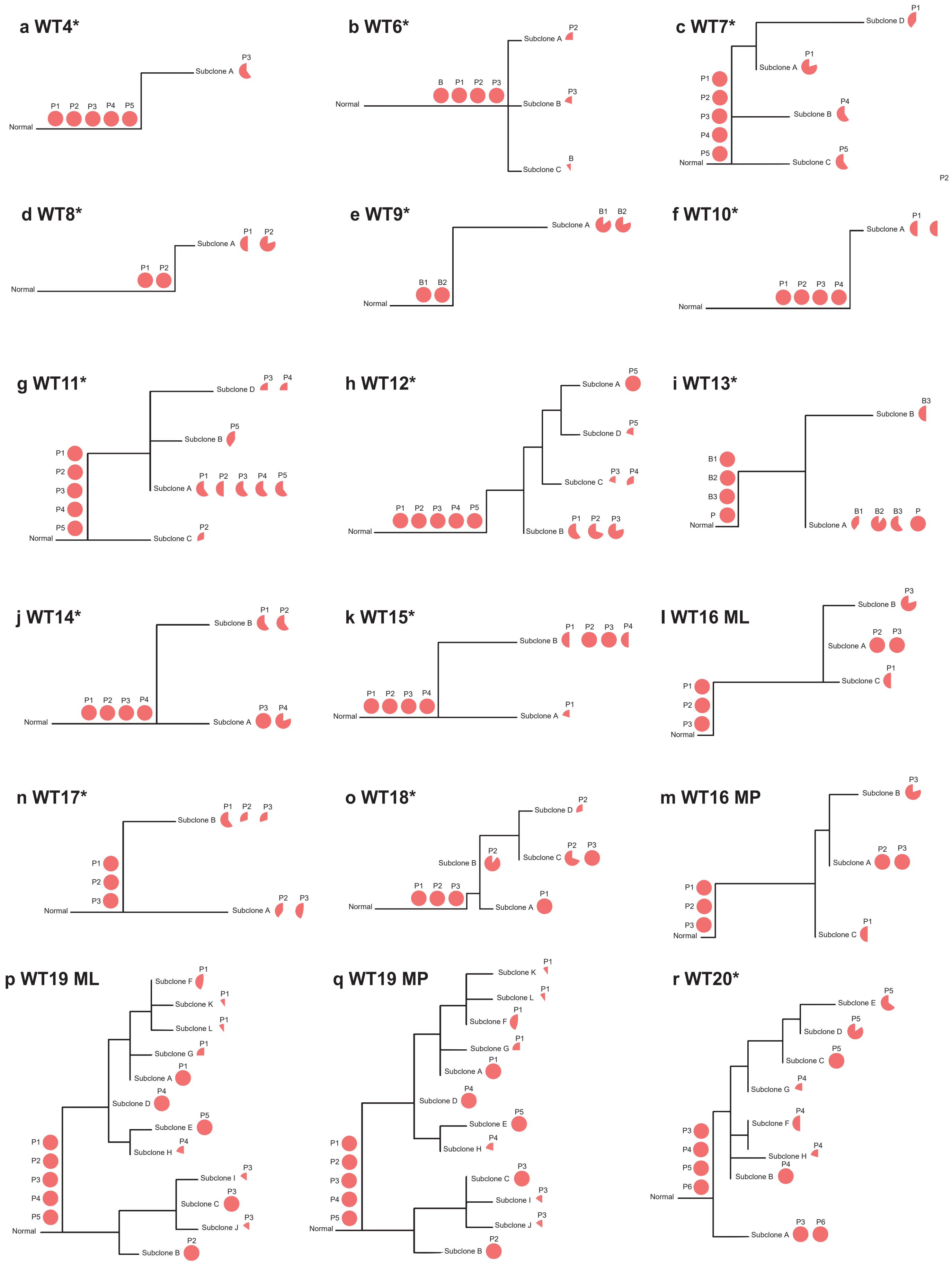

Supplementary Figure 4 Phylogenetic trees for WT4-20. a-r) At the stem, the available biopsies from each patient are visualized by filled pies. An asterix after the patient name indicates that the MP- and ML-trees for this tumor are identical. When they are not identical, both are displayed. The endpoints of the trees represent cell populations harboring distinct genomic profiles (subclones), whose fractions across samples are visualized as pie charts. Biopsies are available from the primary tumor before treatment (B) and after treatment (P), relapses (R), distant metastases (M), lymph node metastases (LN) and lung metastases (LU). The segment files used to produce the phylogenies can be found in Supplementary data 1 and the corresponding event matrices produced by DEVOLUTION in Supplementary data 2.

Supplementary Figure 5

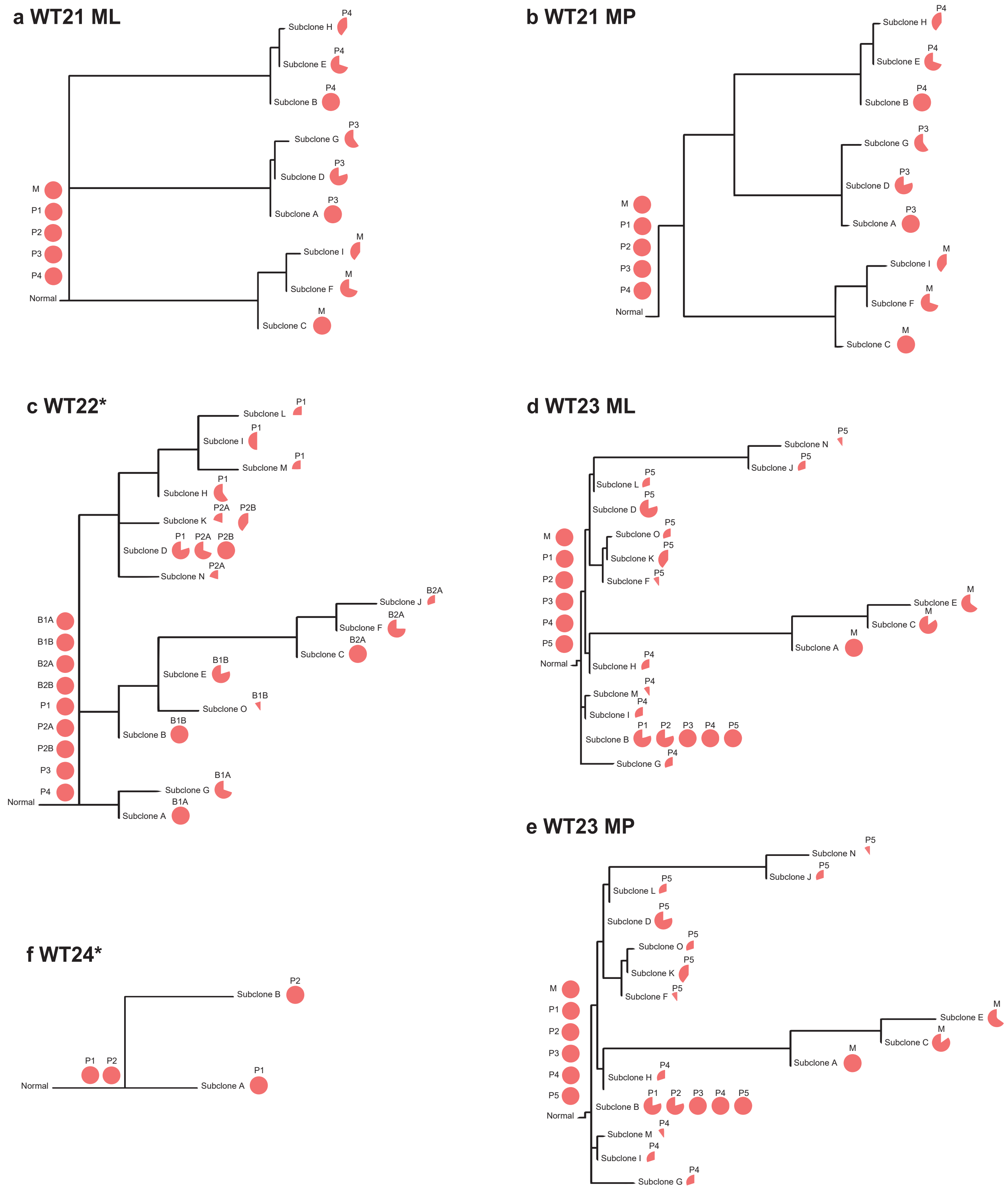

Supplementary Figure 5 Phylogenetic trees for WT 21-24. a-f) At the stem, the available biopsies from each patient are visualized by filled pies. An asterix after the patient name indicates that the MP- and ML-trees for this tumor are identical. When they are not identical, both are displayed. The endpoints of the trees represent cell populations harboring distinct genomic profiles (subclones), whose fractions across samples are visualized as pie charts. Biopsies are available from the primary tumor before treatment (B) and after treatment (P), relapses (R), distant metastases (M), lymph node metastases (LN) and lung metastases (LU). The segment files used to produce the phylogenies can be found in Supplementary data 1 and the corresponding event matrices produced by DEVOLUTION in Supplementary data 2.

Supplementary Figure 6

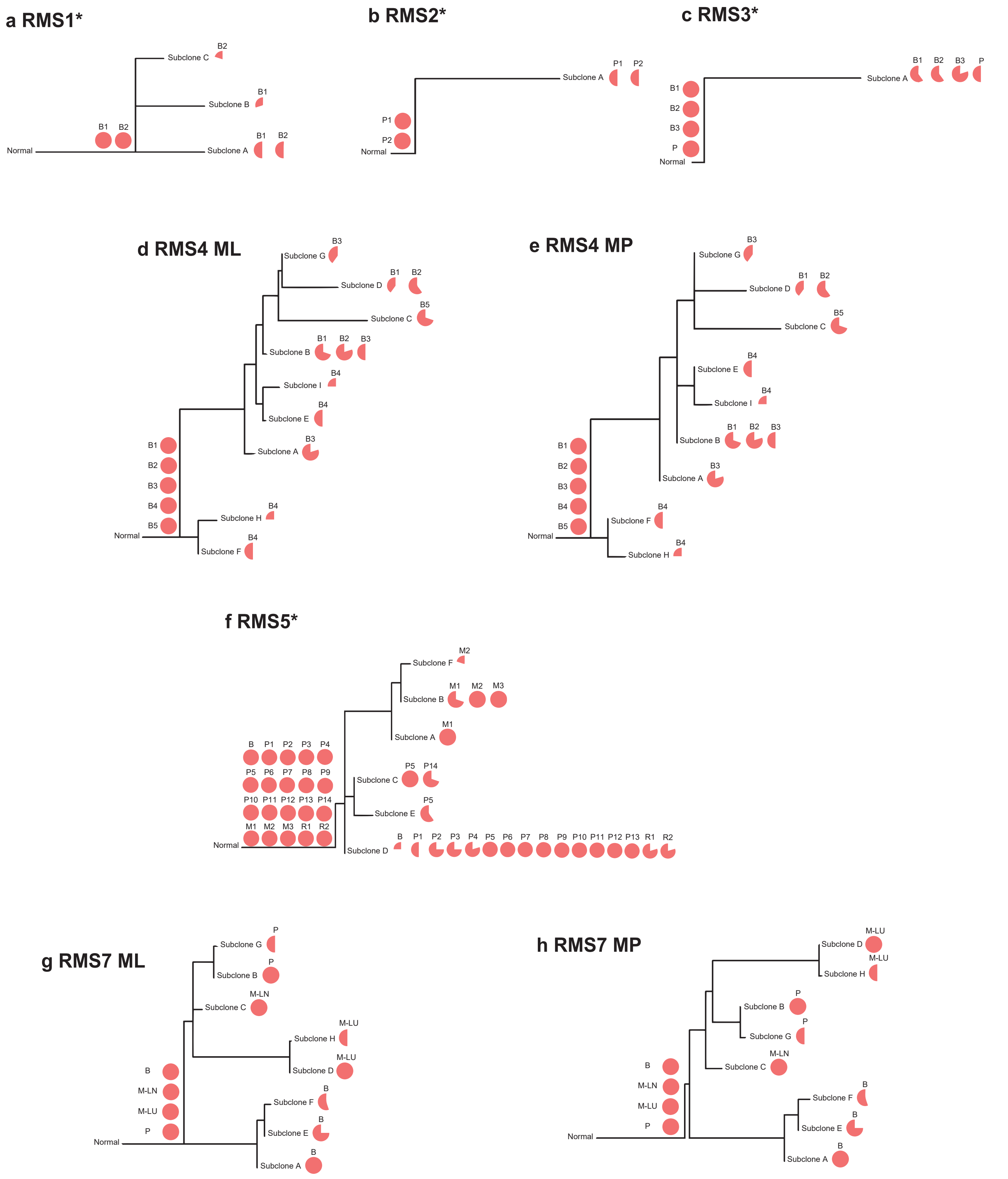

Supplementary Figure 6 Phylogenetic trees for RMS1-5 and 7. a-h) At the stem, the available biopsies from each patient are visualized by filled pies. An asterix after the patient name indicates that the MP- and ML-trees for this tumor are identical. When they are not identical, both are displayed. The endpoints of the trees represent cell populations harboring distinct genomic profiles (subclones), whose fractions across samples are visualized as pie charts. Biopsies are available from the primary tumor before treatment (B) and after treatment (P), relapses (R), distant metastases (M), lymph node metastases (LN) and lung metastases (LU). The segment files used to produce the phylogenies can be found in Supplementary data 1 and the corresponding event matrices produced by DEVOLUTION in Supplementary data 2.

Supplementary Figure 7

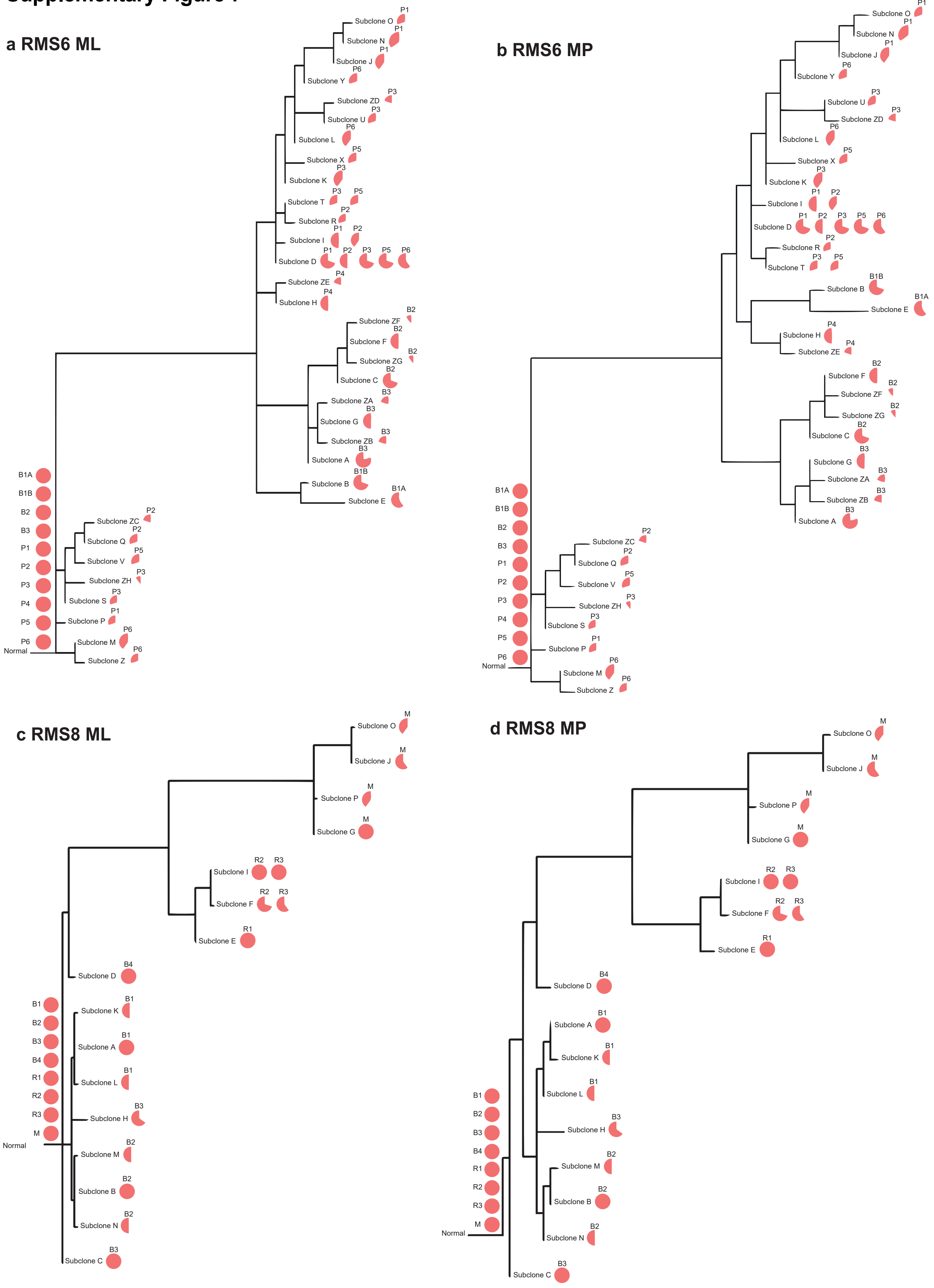

Supplementary Figure 7 Phylogenetic trees for RMS6 and 8. a-d) At the stem, the available biopsies from each patient are visualized by filled pies. An asterix after the patient name indicates that the MP- and ML-trees for this tumor are identical. When they are not identical, both are displayed. The endpoints of the trees represent cell populations harboring distinct genomic profiles (subclones), whose fractions across samples are visualized as pie charts. Biopsies are available from the primary tumor before treatment (B) and after treatment (P), relapses (R), distant metastases (M), lymph node metastases (LN) and lung metastases (LU). The segment files used to produce the phylogenies can be found in Supplementary data 1 and the corresponding event matrices produced by DEVOLUTION in Supplementary data 2.

Supplementary Figure 8

a

Aberration	B1	B2	B3	B4
Stem	100 %	100 %	100 %	100 %
A	-	-	-	100 %
B	-	30 %	90 %	100 %
C	30 %	-	-	-
D	-	-	-	30 %
E	20 %	20 %	30 %	100 %

Aberration	B1	B2	B3	B4
Stem	100 %	100 %	100 %	100 %
Subclone A	-	-	-	100 %
Subclone B	-	30 %	90 %	100 %
Subclone C	30 %	-	-	-
Subclone D	-	-	-	30 %
Subclone E	20 %	20 %	30 %	100 %

	Stem	Sc A	Sc B	Sc C	Sc D	Sc E
Stem	1	1	1	1	1	1
A	0	1	0	0	1	0
B	0	1	1	1	1	0
C	0	0	0	0	0	0
D	0	0	0	0	1	0
E	0	1	0	1	1	1

B1 B2 B3 B4

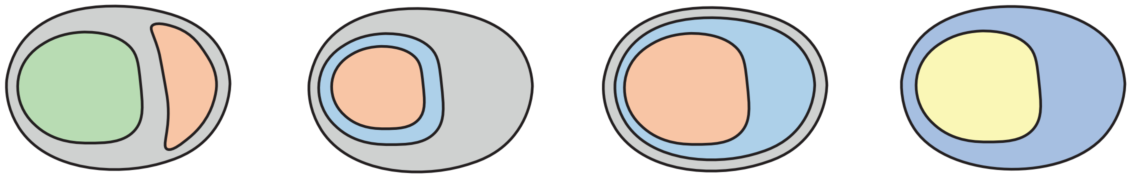

Stem → C
→ E

Stem → B → E

Stem → B → E

Stem → ABE → D

Here, alteration C is seen without alteration B in the sample.

In this sample E seems to originate from the cells having alteration B.

This sample indicated that there actually exist cells containing both aberration B and E.

b

NB14

Tumor ID	Sample ID	Chr	Start	End	Med LogR	VAF (TRS)	Type	Method	Cytoband/ Gene	Clone size (%)		
NB14	ALL	1	44439043.	2,49E+08	NA	NA	GAIN	SNP array	1q21q44 GAIN 1	100	ALL	ALL
NB14	ALL	4	74612636.	1,75E+08	NA	NA	GAIN x5	SNP array	4q34q34 GAIN x5 4	100	ALL	ALL
NB14	ALL	6	102455.0	1,71E+08	NA	NA	GAIN	SNP array	WHOLE GAIN 6	100	ALL	ALL
NB14	ALL	7	20710.5	1,59E+08	NA	NA	GAIN	SNP array	WHOLE GAIN 7	100	ALL	ALL
NB14	ALL	8	86209.0	38484194	NA	NA	LOSS	SNP array	8p23p11 LOSS 8	100	ALL	ALL
NB14	ALL	8	38498637.	43540123	NA	NA	GAIN x3	SNP array	8p11p11 GAIN x3 8	100	ALL	ALL
NB14	ALL	8	25322068.	1,29E+08	NA	NA	GAIN x3	SNP array	8q24q24 GAIN x3 8	100	ALL	ALL
NB14	ALL	8	28538316.	1,46E+08	NA	NA	LOSS	SNP array	8q24q24 LOSS 8	100	ALL	ALL
NB14	ALL	9	9852100.0	10279606	NA	NA	LOSS	SNP array	9p23p23 LOSS 9	100	ALL	ALL
NB14	ALL	17	400959.0	80263427	NA	NA	GAIN	SNP array	WHOLE GAIN 17	100	ALL	ALL
NB14	B1	14	20219083.	1,07E+08	76244997	NA	CNNI	SNP array	WHOLE CNNI 14	100	Subclone_ A	B1 Subclone_ A
NB14	B1	20	69094.0	62912463	035053133	NA	GAIN	SNP array	WHOLE GAIN 20	100	Subclone_ A	B1 Subclone_ A
NB14	B1	8	46896972.	1,25E+08	560939431	NA	GAIN x2	SNP array	8q11q24 GAIN x2 8	60	Subclone_ D	B1 Subclone_ D
NB14	B2	14	20219083.	1,07E+08	22636449	NA	CNNI	SNP array	WHOLE CNNI 14	100	Subclone_ A	B2 Subclone_ A
NB14	B2	20	69094.0	62912463	103915929	NA	GAIN	SNP array	WHOLE GAIN 20	100	Subclone_ A	B2 Subclone_ A
NB14	B2	8	46896972.	1,25E+08	036640999	NA	GAIN x2	SNP array	8q11q24 GAIN x2 8	60	Subclone_ D	B2 Subclone_ D
NB14	B3	14	20511672.	1,07E+08	-0.02	NA	CNNI	SNP array	WHOLE CNNI 14	100	Subclone_ A	B3 Subclone_ A
NB14	B3	20	0.0	62849459	0.15	NA	GAIN	SNP array	WHOLE GAIN 20	100	Subclone_ A	B3 Subclone_ A
NB14	B3	8	46839735.	1,25E+08	0.25	NA	GAIN x2	SNP array	8q11q24 GAIN x2 8	100	Subclone_ D	B3 Subclone_ D
NB14	B3	17	0.0	81027137	0.18	NA	GAIN x2	SNP array	WHOLE GAIN x2 17	60	Subclone_ C	B3 Subclone_ C
NB14	B3	2	0.0	2,43E+08	0.03	NA	GAIN	SNP array	WHOLE GAIN 2	30	Subclone_ E	B3 Subclone_ E
NB14	M	8	46839735.	1,25E+08	0.05	NA	GAIN	SNP array	8q11q24 GAIN 8	100	Subclone_ B	M Subclone_ B
NB14	M	17	0.0	81027137	0.08	NA	GAIN x2	SNP array	WHOLE GAIN x2 17	100	Subclone_ C	M Subclone_ C

B1 B2 B3 M

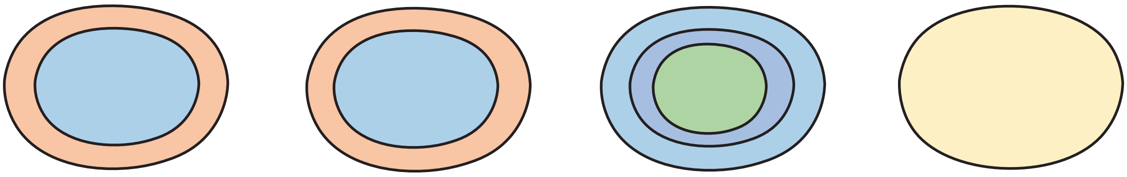

Stem → A → D

Stem → A → D

Stem → AD → C → E

Stem → BC

In this sample alteration C seems to come after D.

This sample C is present without D altogether. Parallel evolution of a whole chromosome aberration may although be correct!

Supplementary Figure 8 Contradictions resulting from parallel evolution or back mutations. a) The leftmost table illustrates the aberrations (first column) in the samples (first row) alongside their fraction in the biopsies. The middle table is the identified clusters and the rightmost table the final event matrix with subclones (Sc) denoted. In biopsy B4 all cells have aberration A, B and E. Hence there exist cells having both B and E. In B2 and B3 alteration E seems to originate from a cell containing the alteration B, but in biopsy B1 alteration E exists without alteration B. The phylogenetic tree can thus not be built from the event matrix without including parallel evolution or back mutations. **b)** An example of a contradiction found in NB14. The table is a segment file as introduced in Supplementary Table 2. In biopsies B1 and B2 all cells have the alterations of cluster A, while approximately 60 % of the cells belong to cluster D, characterized by alteration 8q11q24 gain. In B3 there are cells having alterations A and D, the alteration A, D and C and A, D, C and E. In the metastasis we find alteration B and C in 100 % of the cells. Hence, we here have a case of parallel evolution of tetrasomy 17 (WHOLE GAIN x2). This is actually a biologically plausible event since parallel evolution of whole chromosome aberrations can happen.

Supplementary Figure 9

RMS7

B

7q11q31 LOSS 7	100	Cluster_ A
7q31q31 LOSS 7	100	Cluster_ B
7q31q32 LOSS 7	100	Cluster_ A
7q33q34 LOSS 7	100	Cluster_ A
7q36q36 LOSS 7	100	Cluster_ A
7q36q36v1 LOSS 7	100	Cluster_ A
7q36q36v1v1 LOSS 7	100	Cluster_ A
1q23q32 LOSS 1	100	Cluster_ C
2q37q37 LOSS 2	100	Cluster_ C
2q37q37v1 LOSS 2	100	Cluster_ C
4p15p14 LOSS 4	100	Cluster_ C
5q21q31 LOSS 5	100	Cluster_ C
5q21q31 LOSS 5	100	Cluster_ C
5q35q35 LOSS 5	100	Cluster_ C
5q35q35v1 LOSS 5	100	Cluster_ A
8p21p11 LOSS 8	100	Cluster_ C
9p24p21 LOSS 9	100	Cluster_ C
9p21p21 LOSS 9	100	Cluster_ C
9p21p21v1 LOSS 9	100	Cluster_ C
9q21q21 LOSS 9	100	Cluster_ C
10p15q22 LOSS 10	100	Cluster_ C
10q22q23 LOSS 10	100	Cluster_ C
10q23q23 LOSS 10	100	Cluster_ C
10q23q25 LOSS 10	100	Cluster_ C
10q25q25 LOSS 10	100	Cluster_ C
10q25q26 LOSS 10	100	Cluster_ C
11q14q14 LOSS 11	100	Cluster_ C
11q14q21 LOSS 11	100	Cluster_ C
1q21q22 LOSS 11	100	Cluster_ C
11q22q22 LOSS 11	100	Cluster_ C
11q22q22v1 LOSS 11	100	Cluster_ C
11q22q23 LOSS 11	100	Cluster_ C
11q23q25 LOSS 11	100	Cluster_ C
13q12q12 LOSS 13	100	Cluster_ C
13q12q12v1 LOSS 13	100	Cluster_ C
14q11q12 LOSS 14	100	Cluster_ C
14q13q32 LOSS 14	100	Cluster_ C
17p13p11 LOSS 17	100	Cluster_ C
18q11q12 LOSS 18	100	Cluster_ C
18q12q12 LOSS 18	100	Cluster_ C
18q21q22 LOSS 18	100	Cluster_ C
18q22q23 LOSS 18	100	Cluster_ C
19p13q13 LOSS 19	100	Cluster_ C
WHOLE LOSS 22	100	Cluster_ C
2q13q34 LOSS 2	75	Cluster_ K
2q37q37v1v1 LOSS 2	75	Cluster_ K
8p11p11 LOSS 8	75	Cluster_ K
8p11p11v1 LOSS 8	75	Cluster_ K
19p13p13 LOSS 19	75	Cluster_ K
20p13p11 LOSS 20	75	Cluster_ K
2p25p11 LOSS 2	55	Cluster_ L
2q35q36 LOSS 2	55	Cluster_ L
2q36q36 LOSS 2	55	Cluster_ L
9q22q22 LOSS 9	55	Cluster_ L
12q21q24 LOSS 12	55	Cluster_ L

P

2q13q22 LOSS 2	100	Cluster_ E
3q11q29 LOSS 3	100	Cluster_ E
4q28q31 LOSS 4	100	Cluster_ F
4q34q34 LOSS 4	100	Cluster_ F
13q31q31 LOSS 13	100	Cluster_ E
13q13q13 LOSS 13	100	Cluster_ E
13q31q31v1 LOSS 13	100	Cluster_ E
17p13p11 LOSS 17	100	Cluster_ D
18q11q13 LOSS 19	100	Cluster_ F
1p36p36 LOSS 1	100	Cluster_ F
2p14p14 LOSS 2	100	Cluster_ F
2p11p11 LOSS 2	100	Cluster_ F
2q22q32 LOSS 2	100	Cluster_ G
2q37q37v1v1v1 LOSS 2	100	Cluster_ F
4p14p14 LOSS 4	100	Cluster_ G
7q31q31 LOSS 7	100	Cluster_ B
9p24p21v1 LOSS 9	100	Cluster_ F
13q31q31v1v1 LOSS 13	100	Cluster_ E
18p11p11 LOSS 18	100	Cluster_ G
18q11q11 LOSS 18	100	Cluster_ F
18q21q23 LOSS 18	100	Cluster_ F
Xq22q28 LOSS X	100	Cluster_ F
5p15p14 LOSS 5	50	Cluster_ M
12q24q24 LOSS 12	50	Cluster_ M

M-LN

	2q13q22 LOSS 2	100	Cluster_ E
	3q11q29 LOSS 3	100	Cluster_ E
	4q28q31v1 LOSS 4	100	Cluster_ H
	4q34q35 LOSS 4	100	Cluster_ I
	13q31q31 LOSS 13	100	Cluster_ E
	13q13q13 LOSS 13	100	Cluster_ E
	13q31q31v1 LOSS 13	100	Cluster_ E
*	17p13p11 LOSS 17	100	Cluster_ D
	19q11q13 LOSS 19	100	Cluster_ I
	1p34p31 LOSS 1	100	Cluster_ I
	2q32q37 LOSS 2	100	Cluster_ I
	9p24p13 LOSS 9	100	Cluster_ I
	13q31q31v1v1 LOSS 13	100	Cluster_ E
	21q22q22 LOSS 21	100	Cluster_ I

M-LU

	7q11q31 LOSS 7	100	Cluster_ A
	7q31q31 LOSS 7	100	Cluster_ B
	7q31q32 LOSS 7	100	Cluster_ A
	7q33q34 LOSS 7	100	Cluster_ A
	7q36q36 LOSS 7	100	Cluster_ A
	7q36q36v1 LOSS 7	100	Cluster_ A
	7q36q36v1v1 LOSS 7	100	Cluster_ A
	2q13q22 LOSS 2	100	Cluster_ E
	2q22q32 LOSS 2	100	Cluster_ G
	2q32q32 LOSS 2	100	Cluster_ J
	2q32q32v1 LOSS 2	100	Cluster_ J
	2q32q32v1v1 LOSS 2	100	Cluster_ J
	2q32q32v1v1v1 LOSS 2	100	Cluster_ J
	2q33q33 LOSS 2	100	Cluster_ J
	2q33q33v1 LOSS 2	100	Cluster_ J
	2q33q33v1v1 LOSS 2	100	Cluster_ J
	2q25q35 LOSS 2	100	Cluster_ J
	2q36q36v1 LOSS 2	100	Cluster_ J
	2q36q36v1v1 LOSS 2	100	Cluster_ J
	2q36q36v1v1 LOSS 2	100	Cluster_ J
	2q36q37 LOSS 2	100	Cluster_ J
	3p14p14 LOSS 3	100	Cluster_ J
	3q11q29 LOSS 3	100	Cluster_ E
	4p14p14 LOSS 4	100	Cluster_ G
	4q28q31v1 LOSS 4	100	Cluster_ H
	4q31q34 LOSS 4	100	Cluster_ J
	4q34q35v1 LOSS 4	100	Cluster_ J
	5p15p15 LOSS 5	100	Cluster_ J
	5p15p14v1 LOSS 5	100	Cluster_ J
	5p14p14 LOSS 5	100	Cluster_ J
	5p14p14v1 LOSS 5	100	Cluster_ J
	5p13p13 LOSS 5	100	Cluster_ J
	5p13p13v1 LOSS 5	100	Cluster_ J
	5q35q35v1 LOSS 5	100	Cluster_ A
	8p23p11 LOSS 8	100	Cluster_ J
	9p24p13v1 LOSS 9	100	Cluster_ J
	WHOLE LOSS 10	100	Cluster_ J
	13q31q31 LOSS 13	100	Cluster_ E
	13q13q13 LOSS 13	100	Cluster_ E
	13q31q31v1 LOSS 13	100	Cluster_ E
	13q31q31v1v1 LOSS 13	100	Cluster_ E
	13q34q34 LOSS 13	100	Cluster_ J
	14q11q12v1 LOSS 14	100	Cluster_ J
	14q13q32v1 LOSS 14	100	Cluster_ J
	15q23q23 LOSS 15	100	Cluster_ J
	15q24q24 LOSS 15	100	Cluster_ J
	15q24q24v1 LOSS 15	100	Cluster_ J
	17p13p13 LOSS 17	100	Cluster_ J
*	17p13p11v1 LOSS 17	100	Cluster_ J
	17q22q22 LOSS 17	100	Cluster_ J
	17q22q23 LOSS 17	100	Cluster_ J
	17q24q24 LOSS 17	100	Cluster_ J
	18p11p11 LOSS 18	100	Cluster_ G
	19q13q13 LOSS 19	100	Cluster_ J
	20p13p11v1 LOSS 20	100	Cluster_ J
	20p13p13 LOSS 20	100	Cluster_ J
	20p11p11 LOSS 20	100	Cluster_ J
	5q11q35 LOSS 5	50	Cluster_ N

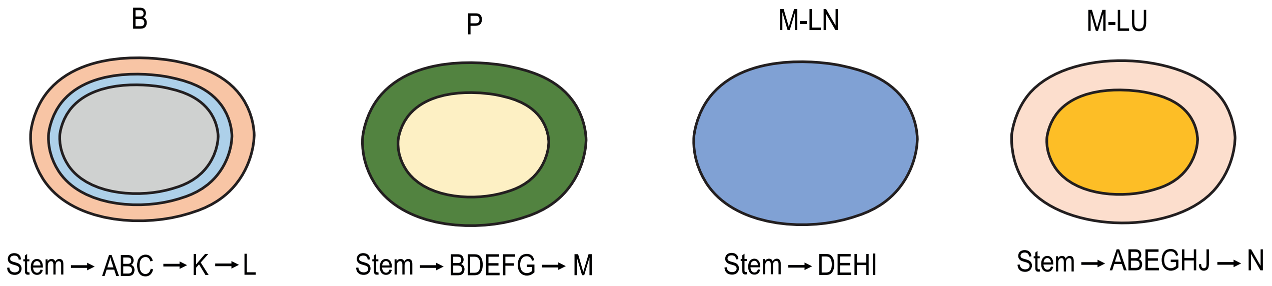

Alteration B exist in a cell also having A and C.

Alteration B exist in a cell also having DEFG. This can be true if B is an early event originating before DEFG in time. The absence of A and C also indicates that they are later events than B.

The alterations in cluster D and E are present without B.

The user might want to reconsider if one of the events in cluster J actually is D (*).

Supplementary Figure 9 Contradictions in a complex dataset. The tables illustrate the alterations in RMS7 found in biopsy before treatment (B), after treatment (P), a lymph node metastasis (M-LN), and a lung metastasis (M-LU). The first column is the location and type of alteration, the second column its size in the biopsy and the third its clustering. The ovals in the lower part of the figure illustrates the suggested temporal allocation of the clusters of events based on their sizes across the biopsies. In this case all events are > 50 % resulting in only one solution to how the clones are nested. Below each oval the temporal allocation is indicated. In biopsy B the alteration in cluster B exist in a cell having the alterations contained by clusters A and C. In B it exists in a cell having the alteration contained by clusters D, E, F and G. From these two biopsies from the primary tumor it thus seems as if B is a very early event in the evolution of the tumor. When looking at the lymph node biopsy we find cells having the alterations contained by D, E, H and I without B. This indicates that B is lost, which is unlikely since it is an intrachromosomal aberration. In addition, one of the alterations in cluster J might actually represent the same genetic alteration as the one forming cluster D in the other samples. It is considered to be two different events in this example since one of the end points differ by more than 1 Mbp. Reconsidering these two alterations results in a tree without contradictions in the evolutionary history of this rhabdomyosarcoma.

Supplementary Figure 10

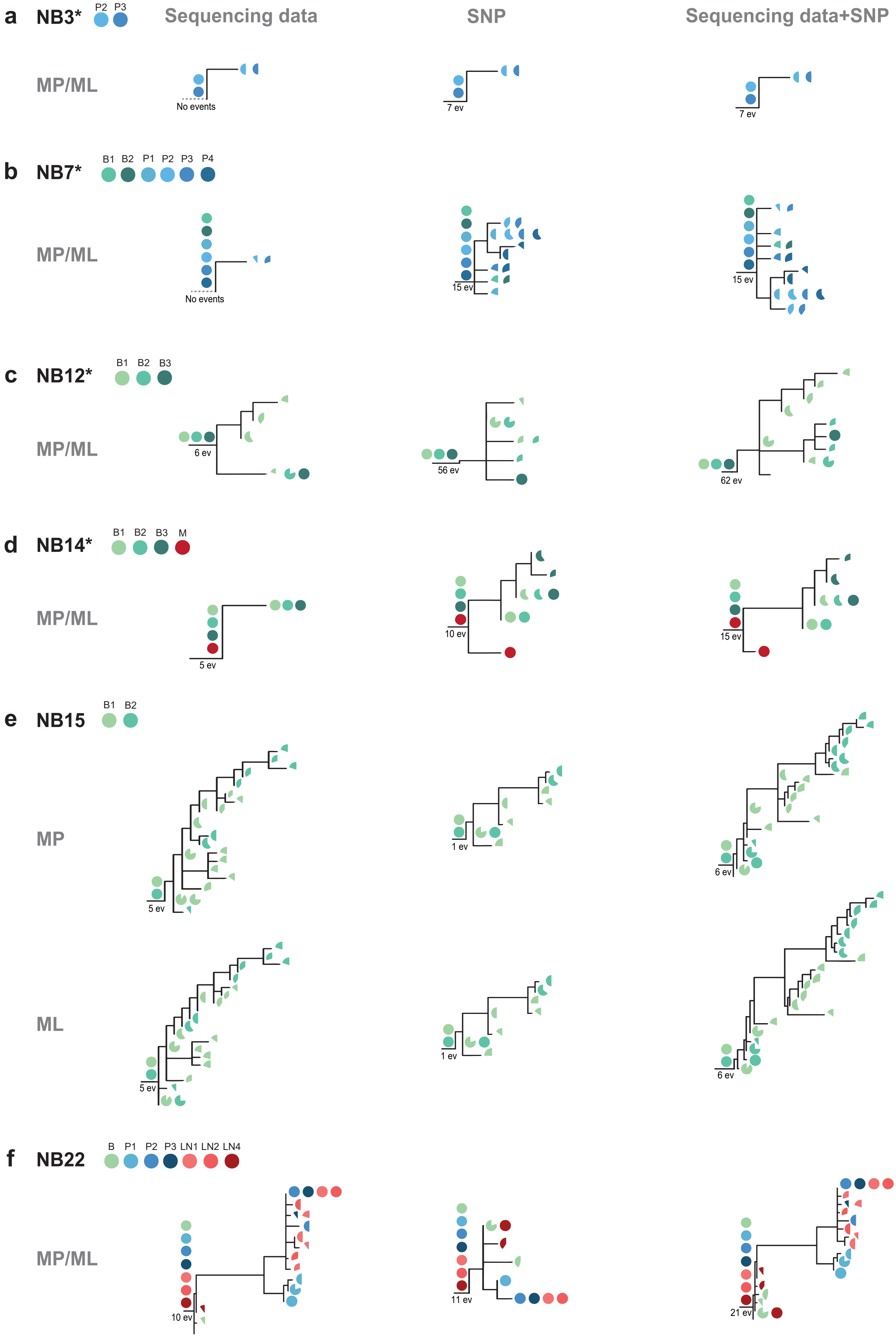

Supplementary Figure 10 comparison between phylogenetic trees based on sequencing data alone, SNP-array data alone and when they are used in unison. a-f) Phylogenetic trees constructed for 6 NB. At the stem, the number of events is annotated as well as the available biopsies from each by filled pies of differing colors. An asterix after the tumor name indicates that the MP- and ML-trees for this tumor are identical. When they are not identical, both are displayed for which the upper row represents MP-trees and the bottom row the ML-trees. The endpoints of the trees represent cell populations harboring distinct genomic profiles (subclones), whose fractions across samples are visualized as pie charts. Biopsies are available from the primary tumor before treatment (B) and after treatment (P), relapses (R), distant metastases (M), lymph node metastases (LN) and lung metastases (LU). The information used to produce the phylogenies can be found in Supplementary data 5.

Supplementary Figure 11

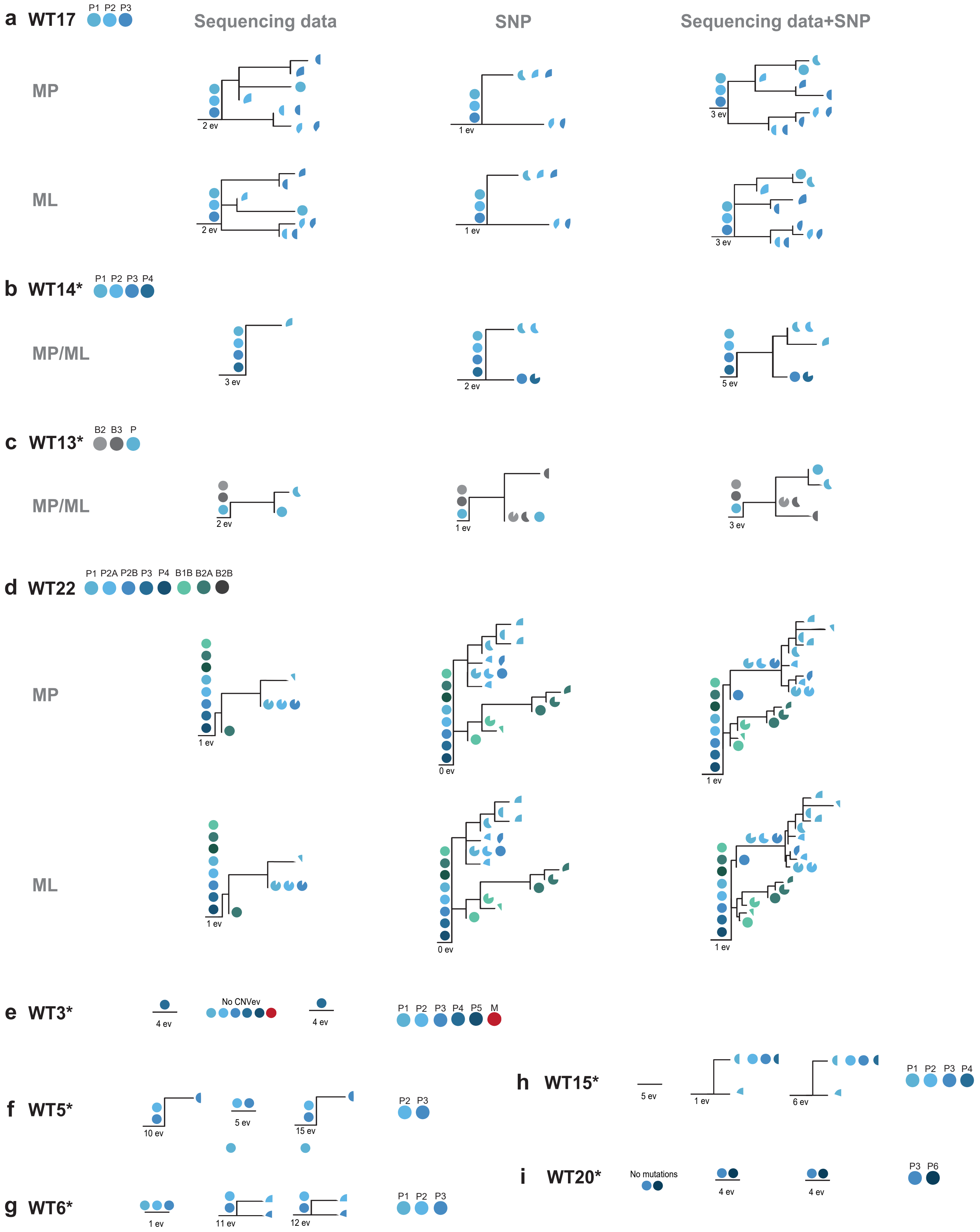

Supplementary Figure 11 comparison between phylogenetic trees based on sequencing data alone, SNP-array data alone and when they are used in unison. a-i) Phylogenetic trees constructed for 9 WT. At the stem, the number of events is annotated as well as the available biopsies from each by filled pies of differing colors. An asterix after the tumor name indicates that the MP- and ML-trees for this tumor are identical. When they are not identical, both are displayed for which the upper row represents MP-trees and the bottom row the ML-trees. The endpoints of the trees represent cell populations harboring distinct genomic profiles (subclones), whose fractions across samples are visualized as pie charts. Biopsies are available from the primary tumor before treatment (B) and after treatment (P), relapses (R), distant metastases (M), lymph node metastases (LN) and lung metastases (LU). The information used to produce the phylogenies can be found in Supplementary data 5.

Supplementary Figure 12

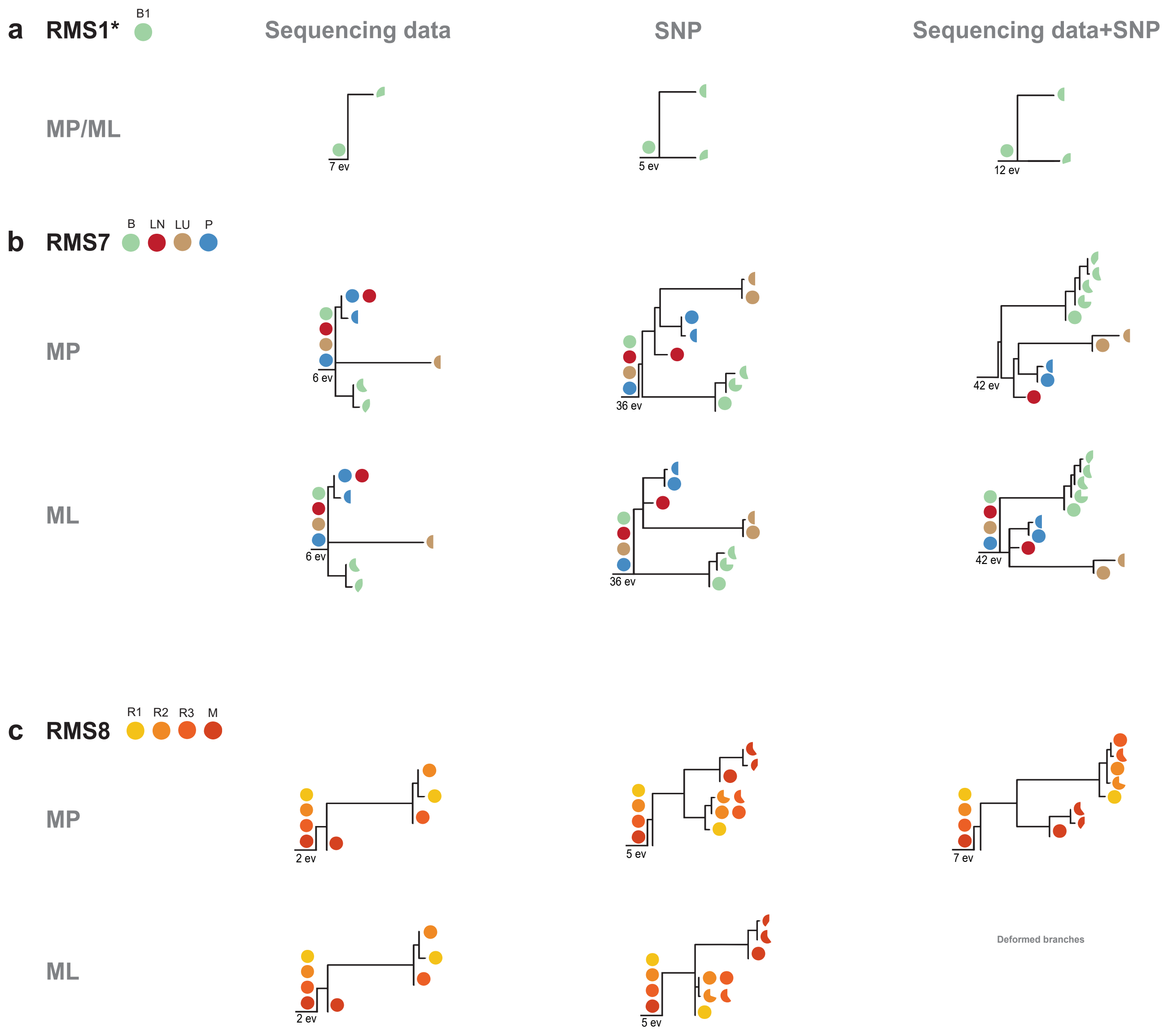

Supplementary Figure 12 comparison between phylogenetic trees based on sequencing data alone, SNP-array data alone and when they are used in unison. a-c) Phylogenetic trees constructed for 3 RMS. At the stem, the number of events is annotated as well as the available biopsies from each by filled pies of differing colors. An asterix after the tumor name indicates that the MP- and ML-trees for this tumor are identical. When they are not identical, both are displayed for which the upper row represents MP-trees and the bottom row the ML-trees. The endpoints of the trees represent cell populations harboring distinct genomic profiles (subclones), whose fractions across samples are visualized as pie charts. Biopsies are available from the primary tumor before treatment (B) and after treatment (P), relapses (R), distant metastases (M), lymph node metastases (LN) and lung metastases (LU). The information used to produce the phylogenies can be found in Supplementary data 5.

Supplementary Figure 13

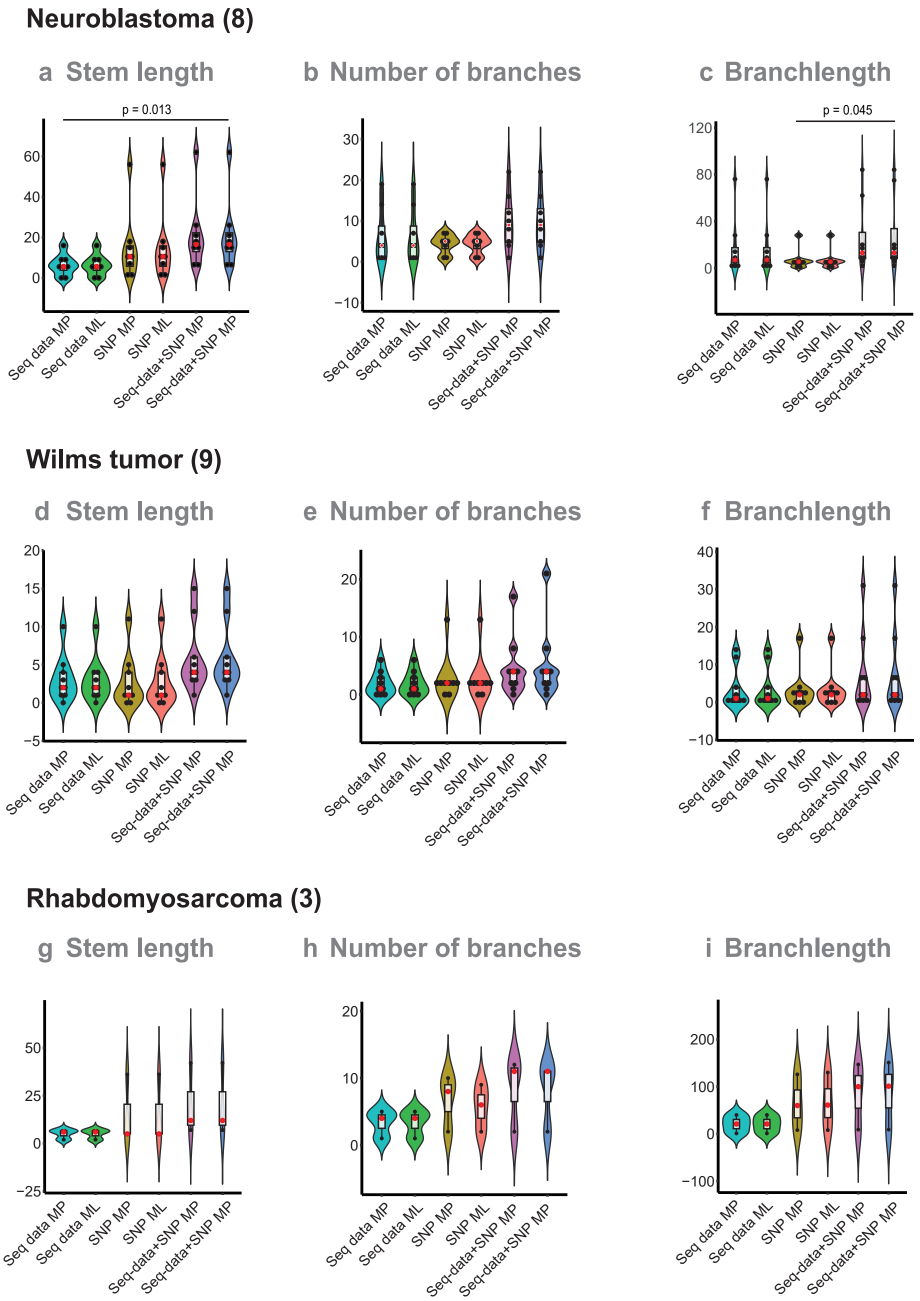

Supplementary Figure 13 Violin plots for the comparison between phylogenetic trees based on sequencing data alone, SNP-array data alone and when they are used in unison. a-i) Violin plots for the stem length, number of branches and branch length for the phylogenetic trees constructed based on sequencing data alone, SNP-array data alone and when the two datasets were used in unison. Significant differences are annotated in the figure. P-values were computed using a two-sided Mann-Whitney U-test. The box plots within the violin plots illustrate the interquartile range. The red dot is the median value. The information used to produce the phylogenies can be found in Supplementary data 5.

Supplementary Figure 14

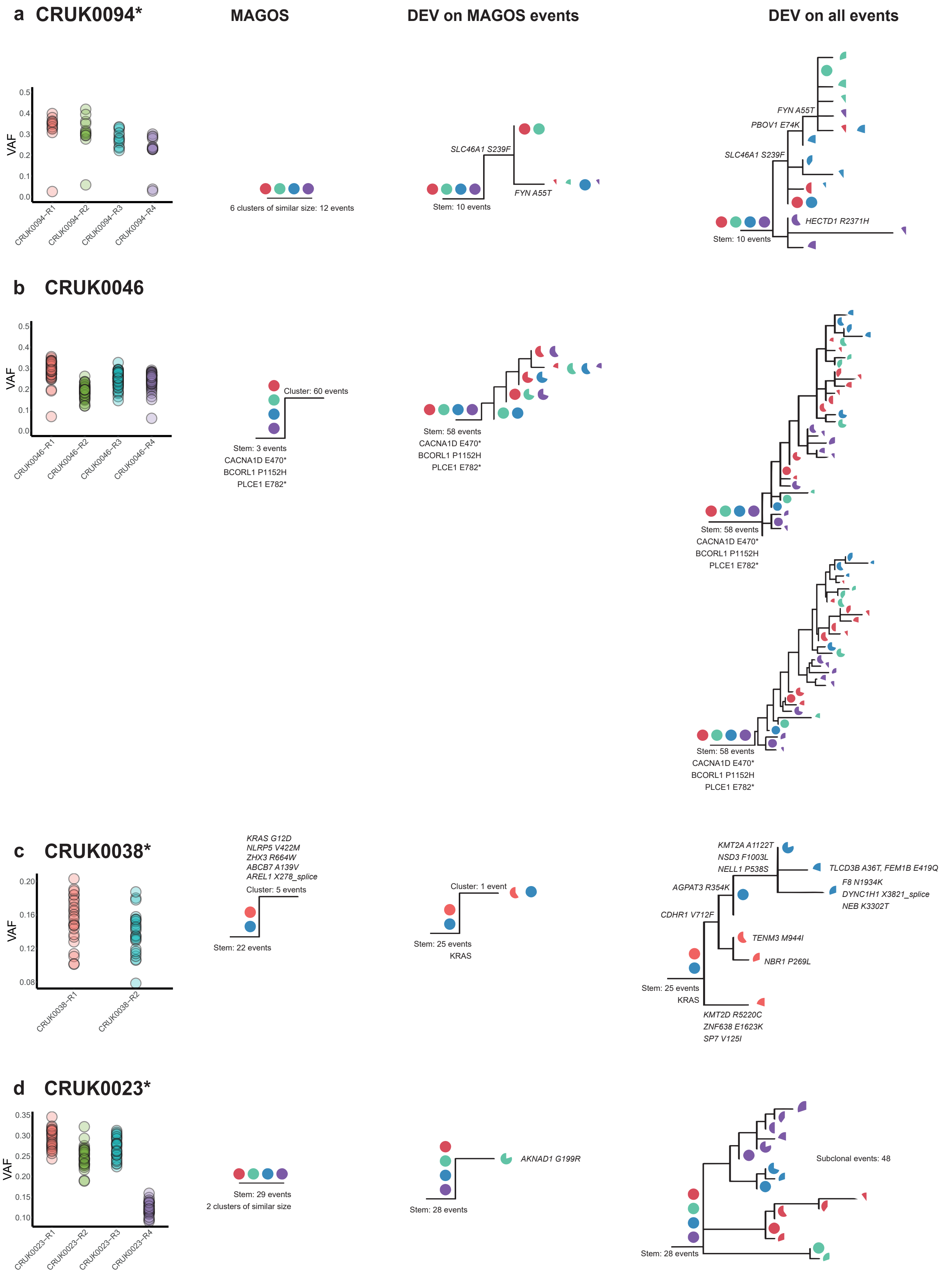

Supplementary Figure 14 TRACERx analysis using DEVOLUTION and MAGOS. a-d) Result of the analysis of the 20 NSCLC from the TRACERx data set. The leftmost scatterplot illustrates the VAF-distribution of the events that passed quality control across the biopsies. Above the plot the corresponding tumor name is denoted. An asterisk indicates that the MP- and ML-trees were identical. When they are not identical, both are displayed, for which the upper row represents MP-trees and the bottom row the ML-trees. The leftmost phylogenetic tree is based on manual nesting of the clusters obtained using MAGOS. The middle phylogenetic tree is the output of DEVOLUTION on the events feasible for analysis with MAGOS i.e. solely events present in all biopsies. The rightmost phylogenetic tree is the output of DEVOLUTION based on all mutations that passed quality control, i.e. also events that are found in merely a subset of samples. In CRUK0068 the MAGOS clustering results in 5 clusters denoted cluster 2-6. The clusters cross each other in size across the sizes, which makes it not feasible to do a nesting that are in concordance across all biopsies. The information used to produce the phylogenies can be found in Supplementary data 6.

Supplementary Figure 15

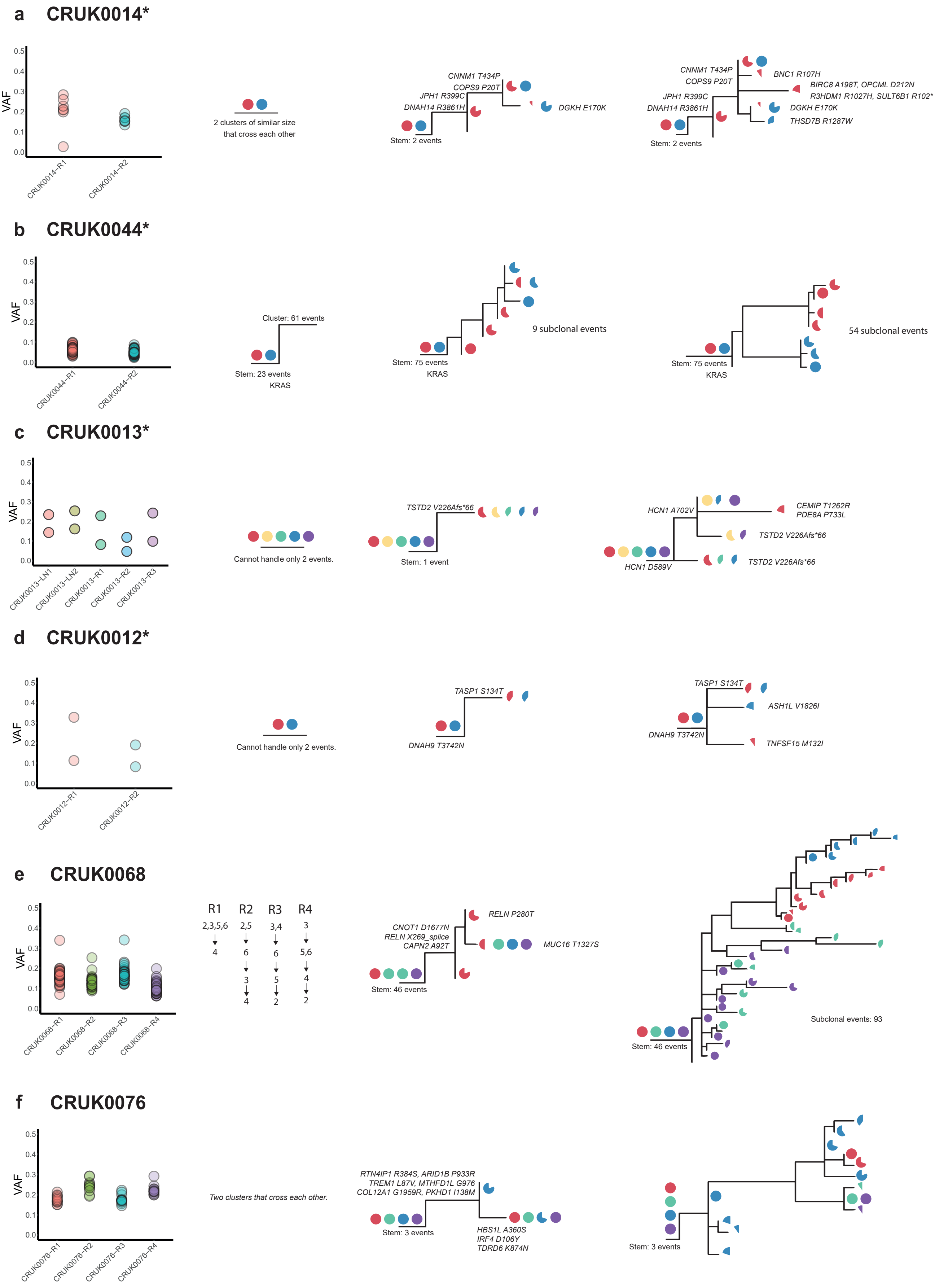

Supplementary Figure 15 TRACERx analysis using DEVOLUTION and MAGOS. a-f) Result of the analysis of the 20 NSCLC from the TRACERx data set. The leftmost scatterplot illustrates the VAF-distribution of the events that passed quality control across the biopsies. Above the plot the corresponding tumor name is denoted. An asterisk indicates that the MP- and ML-trees were identical. When they are not identical, both are displayed, for which the upper row represents MP-trees and the bottom row the ML-trees. The leftmost phylogenetic tree is based on manual nesting of the clusters obtained using MAGOS. The middle phylogenetic tree is the output of DEVOLUTION on the events feasible for analysis with MAGOS i.e. solely events present in all biopsies. The rightmost phylogenetic tree is the output of DEVOLUTION based on all mutations that passed quality control, i.e. also events that are found in merely a subset of samples. The information used to produce the phylogenies can be found in Supplementary data 6.

Supplementary Figure 16

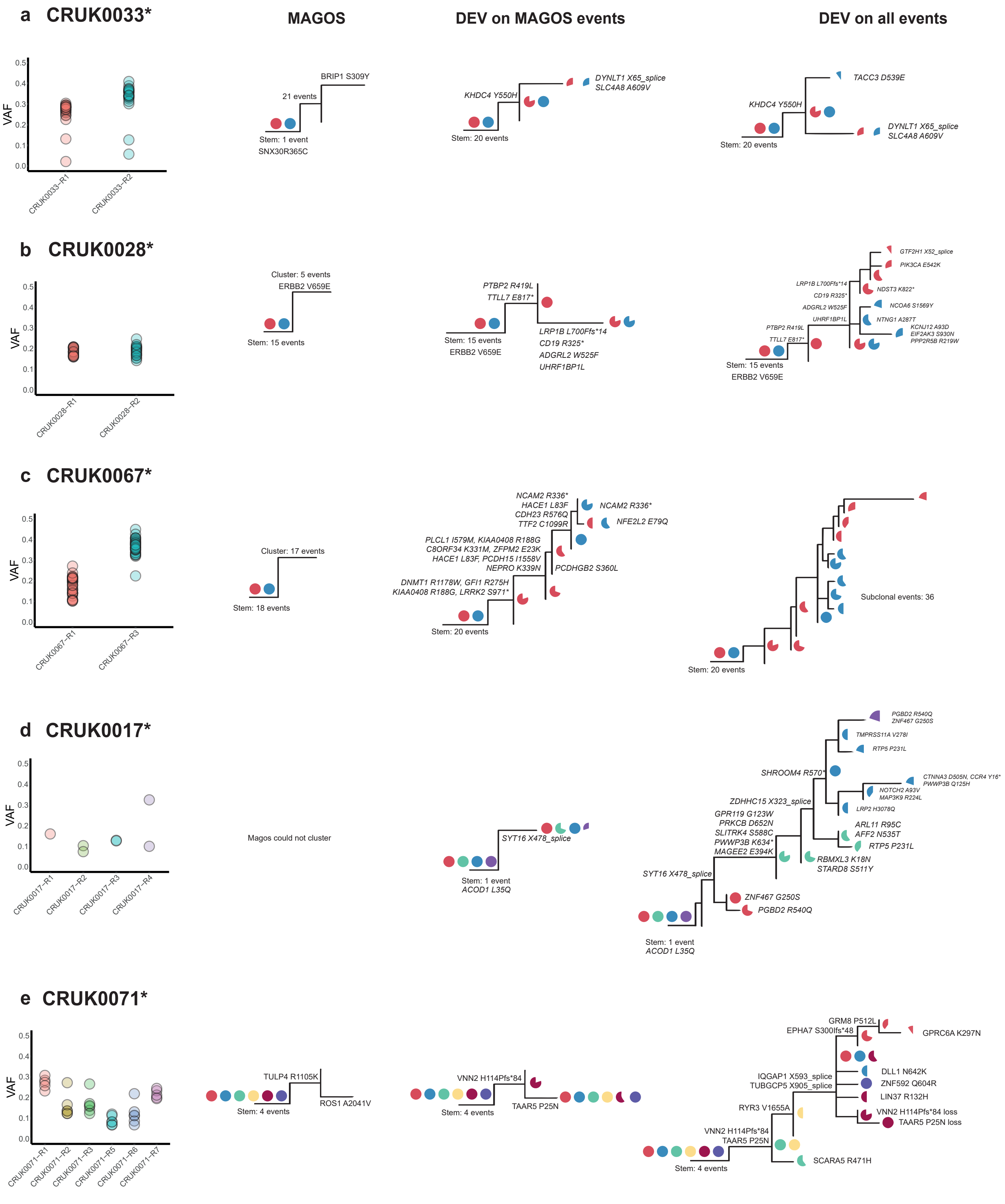

Supplementary Figure 16 TRACERx analysis using DEVOLUTION and MAGOS. a-e) Result of the analysis of the 20 NSCLC from the TRACERx data set. The leftmost scatterplot illustrates the VAF-distribution of the events that passed quality control across the biopsies. Above the plot the corresponding tumor name is denoted. An asterisk indicates that the MP- and ML-trees were identical. When they are not identical, both are displayed, for which the upper row represents MP-trees and the bottom row the ML-trees. The leftmost phylogenetic tree is based on manual nesting of the clusters obtained using MAGOS. The middle phylogenetic tree is the output of DEVOLUTION on the events feasible for analysis with MAGOS i.e. solely events present in all biopsies. The rightmost phylogenetic tree is the output of DEVOLUTION based on all mutations that passed quality control, i.e. also events that are found in merely a subset of samples. The information used to produce the phylogenies can be found in Supplementary data 6.

Supplementary Figure 17

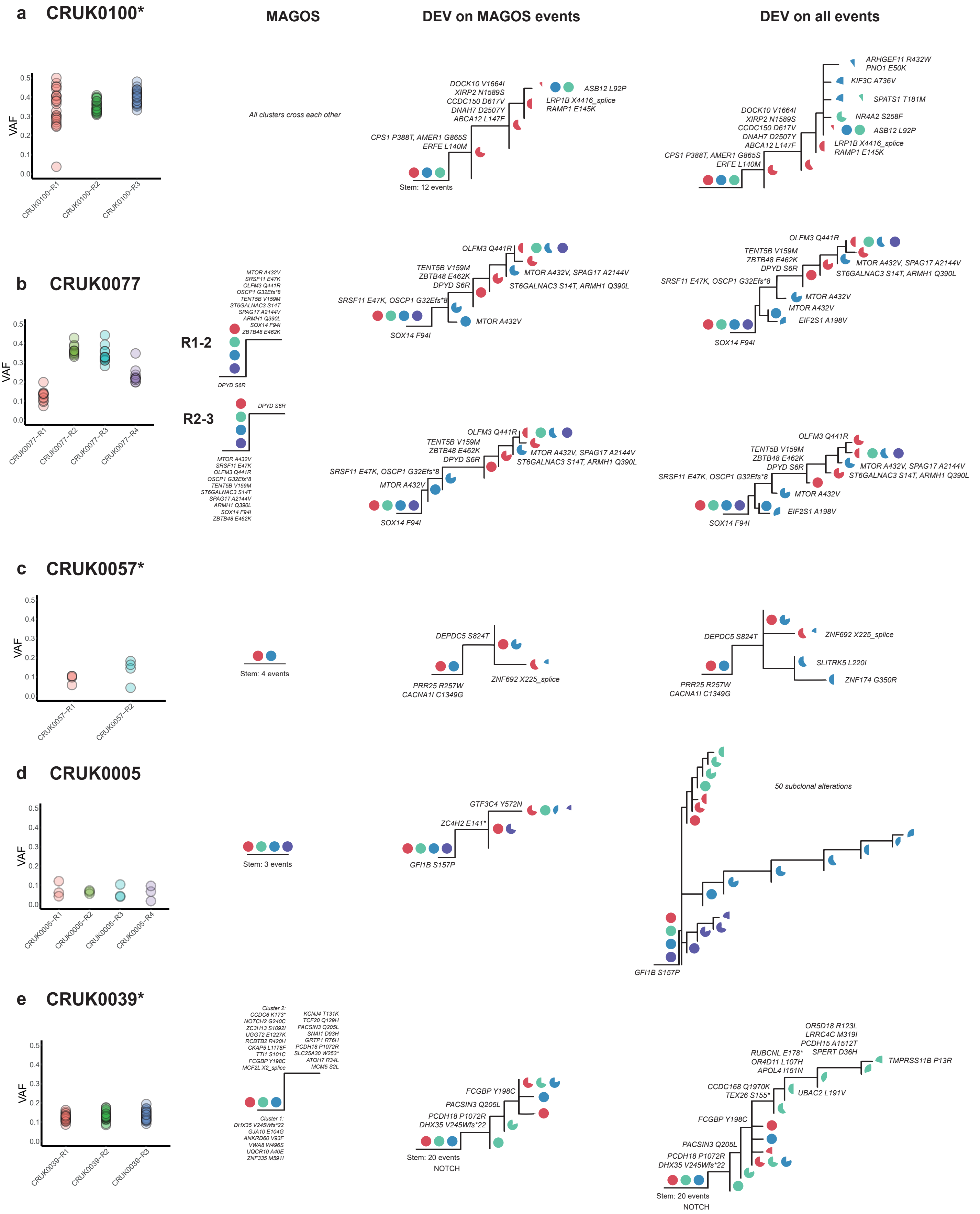

Supplementary Figure 17 TRACERx analysis using DEVOLUTION and MAGOS. a-e) Result of the analysis of the 20 NSCLC from the TRACERx data set. The leftmost scatterplot illustrates the VAF-distribution of the events that passed quality control across the biopsies. Above the plot the corresponding tumor name is denoted. An asterisk indicates that the MP- and ML-trees were identical. When they are not identical, both are displayed, for which the upper row represents MP-trees and the bottom row the ML-trees. The leftmost phylogenetic tree is based on manual nesting of the clusters obtained using MAGOS. The middle phylogenetic tree is the output of DEVOLUTION on the events feasible for analysis with MAGOS i.e. solely events present in all biopsies. The rightmost phylogenetic tree is the output of DEVOLUTION based on all mutations that passed quality control, i.e. also events that are found in merely a subset of samples. The information used to produce the phylogenies can be found in Supplementary data 6.

Supplementary Figure 18

a

	S1	S2
**Mut 1**	100	100
**Mut 2**	60	80
**Mut 3**	30	80
**Mut 4**	10	20

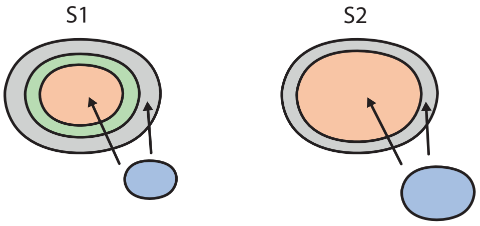

b

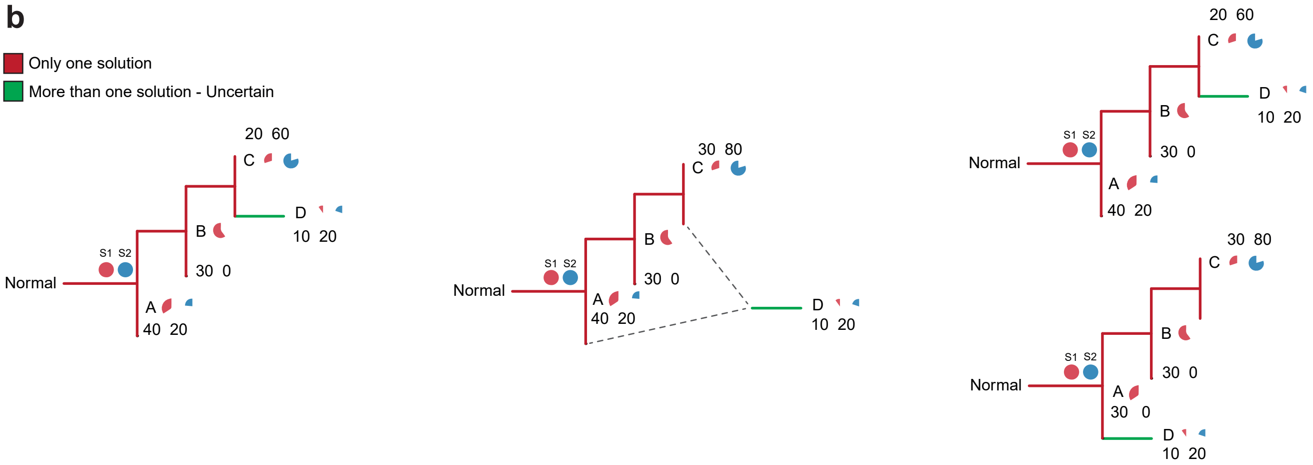

Supplementary Figure 18 Assessing alternative solutions. a) An example of what the algorithm ”subclones” does (flowchart in S. Figure 25). Imagine that we have 2 samples, S1 and S2. In each sample 4 mutations (mut) have been found whose MCF:s are visualized in the table. When nesting the clusters there are two possible nesting patterns for mutation 4. **b)** The leftmost phylogeny is the one suggested by DEVOLUTION. The subclones including clusters of genetic alterations with multiple nesting patterns are visualized with green branches while the ones only having a single solution are red. The numbers in the phylogeny denote the space left at each level in the tree. The algorithm removes the subclones having multiple solutions from the phylogenetic tree structure, leaving only the part of the phylogeny that there is a single solution for as can be seen in the middle phylogeny, also altering the spaces left at each level. A new solution is randomly selected. Here there are only two solutions, visualized as the rightmost two phylogenies.

Supplementary Figure 19 Function: load_matrix

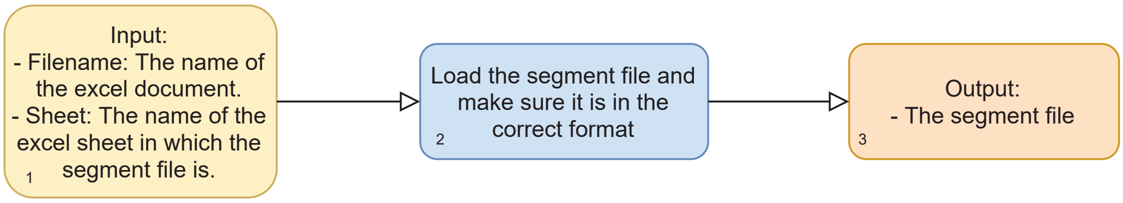

Supplementary Figure 19 Flow chart for the function load_matrix. The input data file should be in the same format as visualized in Supplementary Table 2. The function loads the matrix and makes sure it is in the correct format.

Supplementary Figure 20 Function: splitdata

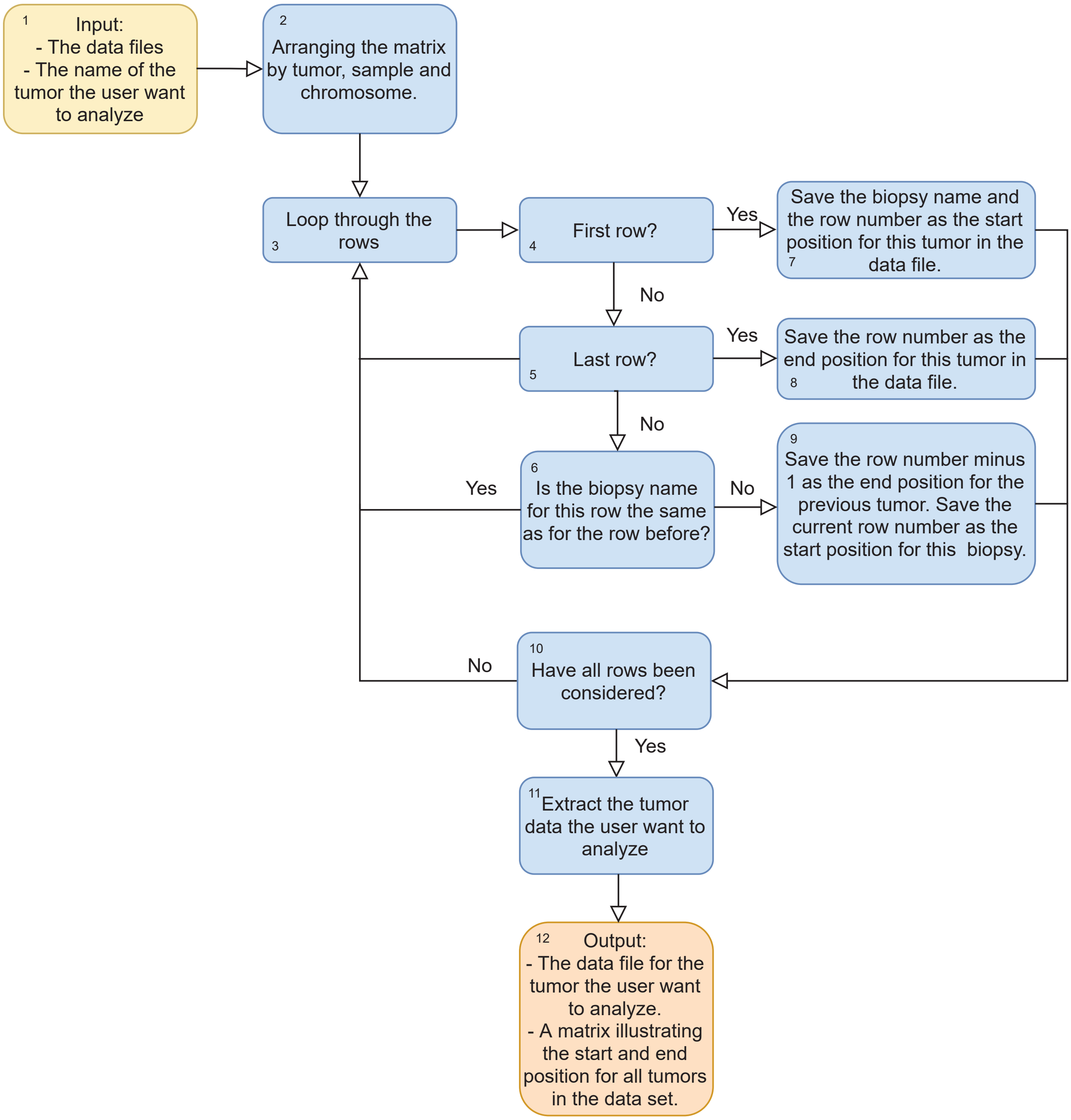

Supplementary Figure 20 Flow chart for the function splitdata. The output from the algorithm in S. Figure 59 is used as an input to this algorithm along with information about which tumor the user wants to analyze. The algorithm extracts the part of the input file that belongs to this tumor. It also provides the user with information about the start and location of all unique tumors included in the input data file.

Supplementary Figure 21 **Function: DEVOLUTION**

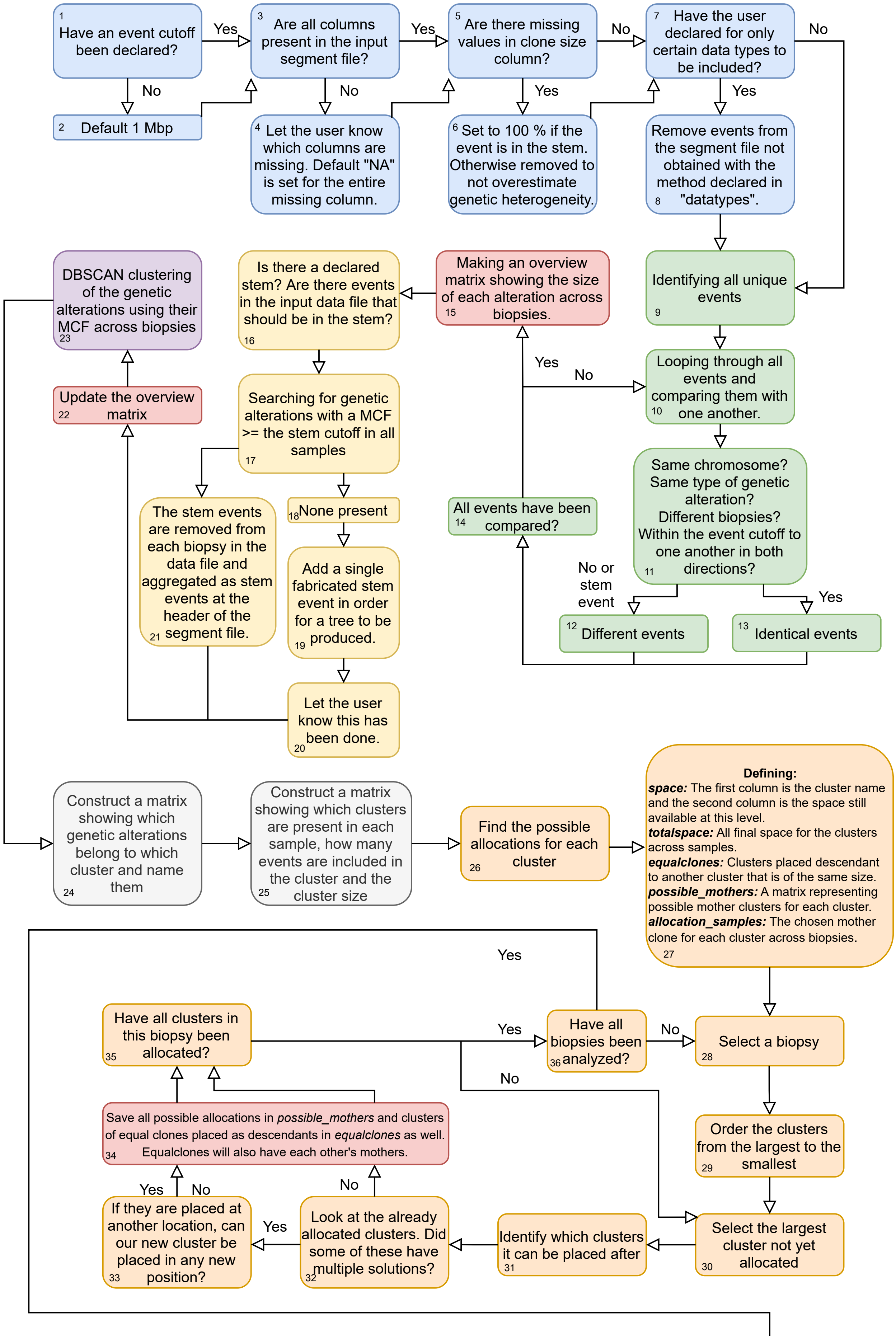

Supplementary Figure 21 The DEVOLUTION algorithm. A flow chart of the DEVOLUTION algorithm. Initually preprocessing of the input data file is done (box 1-8). Then unique events across the samples are identified (box 9-14) using the principle described in Supplementary Figure 1. The stem is identified (box 15-22). The genetic alterations are clustered based on their pattern across biopsies using the DBSCAN algorithm (box 23-25). They an algorithm identifies all the possible nestings for each of the identified clusters of genetic alterations (box 26-36).

Supplementary Figure 22

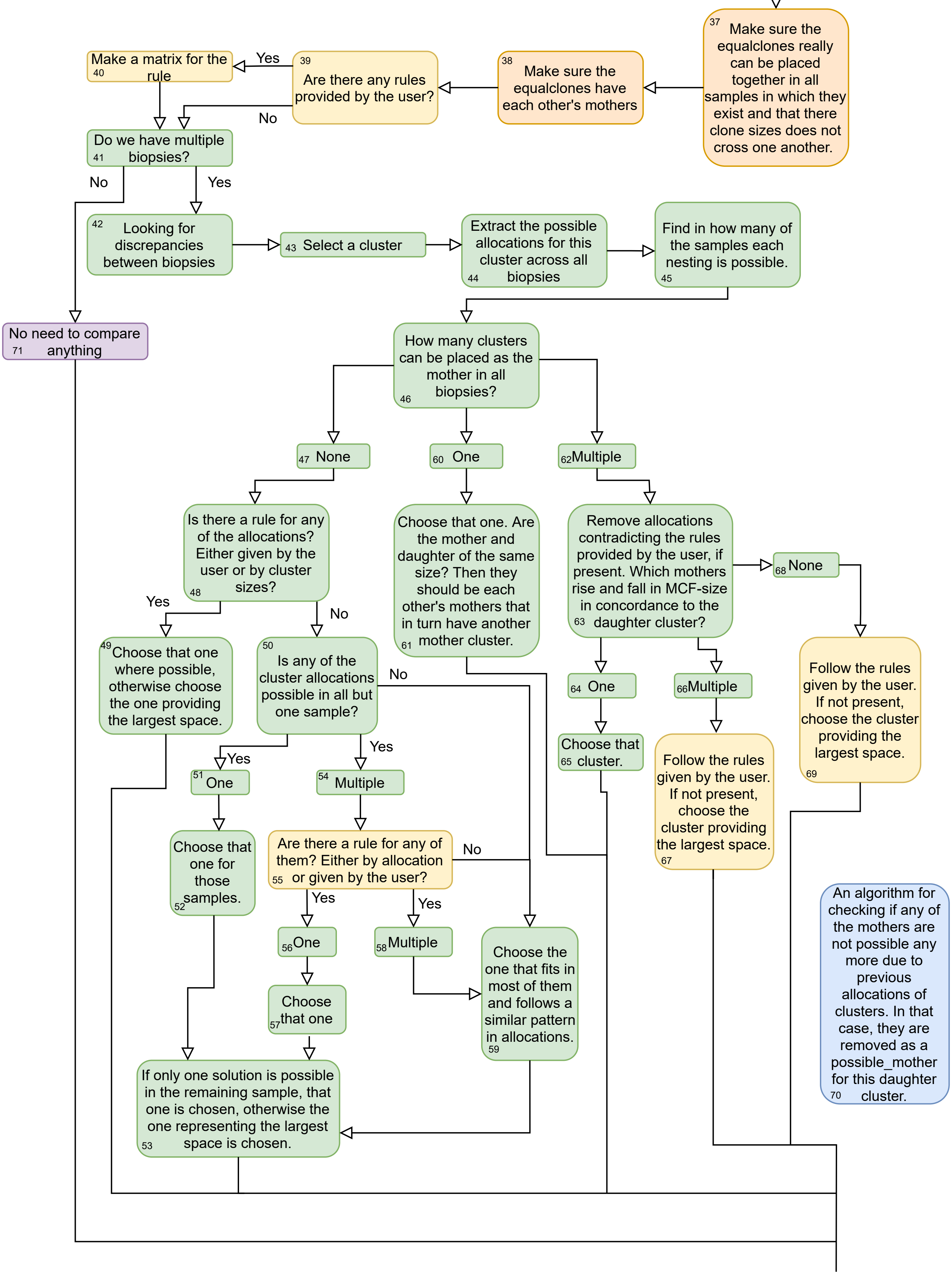

Supplementary Figure 22 The DEVOLUTION algorithm. A flow chart of the DEVOLUTION algorithm. After identifying the possible nestings of the clusters of genetic mutations across samples, an algorithm searches for solutions of the nesting that are feasible across all samples in which the clusters appear in (box 37-71).

Supplementary Figure 23

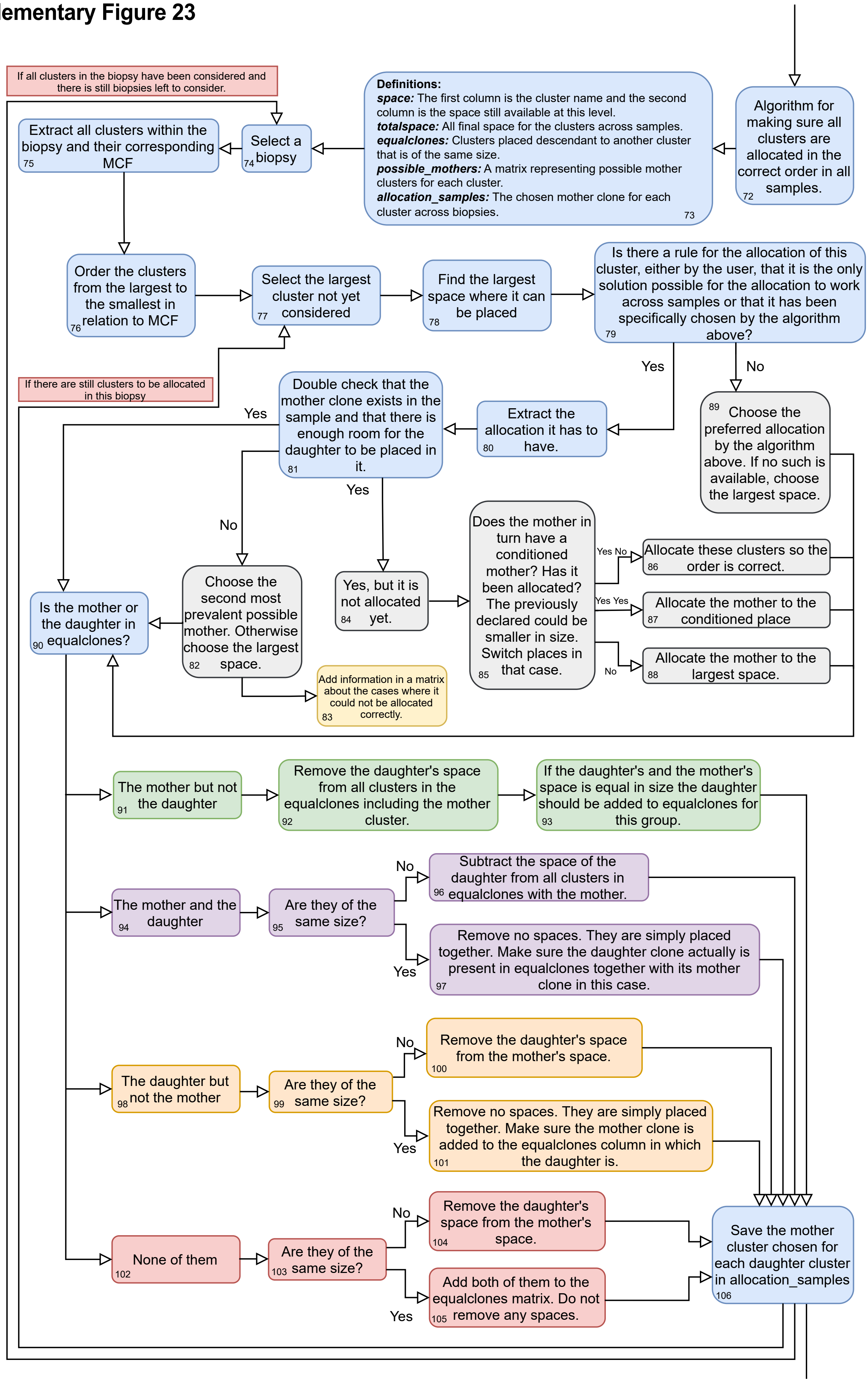

Supplementary Figure 23 The DEVOLUTION algorithm. A flow chart of the DEVOLUTION algorithm. After identifying the possible nestings that are in concordance with as many of the samples as possible, the nesting is made. During the nesting, rules provided from the user concerning illicit biological orders are also taken into consideration (box 72-106).

Supplementary Figure 24

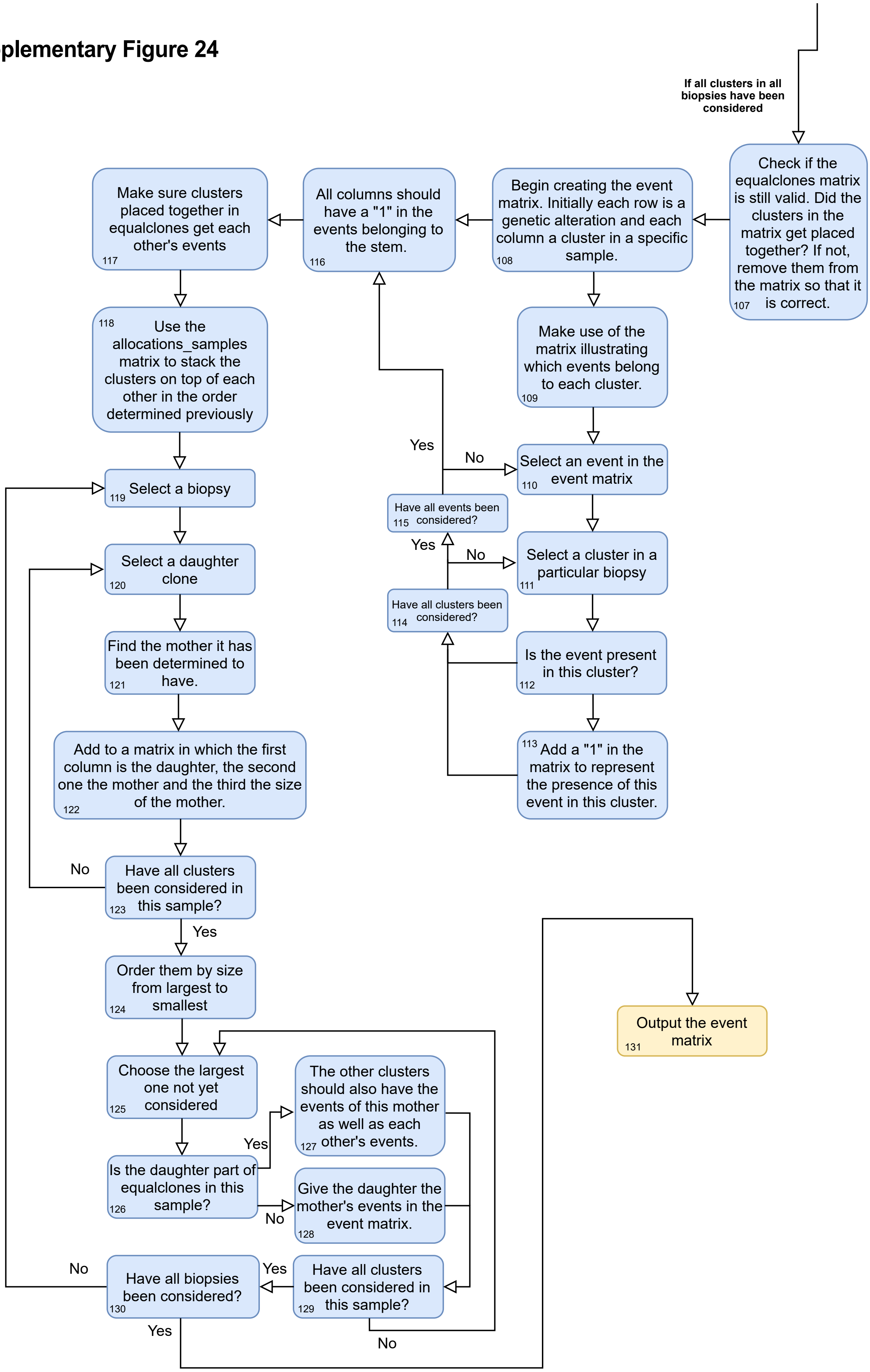

Supplementary Figure 24 The DEVOLUTION algorithm. A flow chart of the DEVOLUTION algorithm. When the nesting of the clusters have been made in a way that is in concordance with as many of the samples as possible, the event matrix is constructed. This event matrix is the output of the algorithm (box 107-131).

Supplementary Figure 25

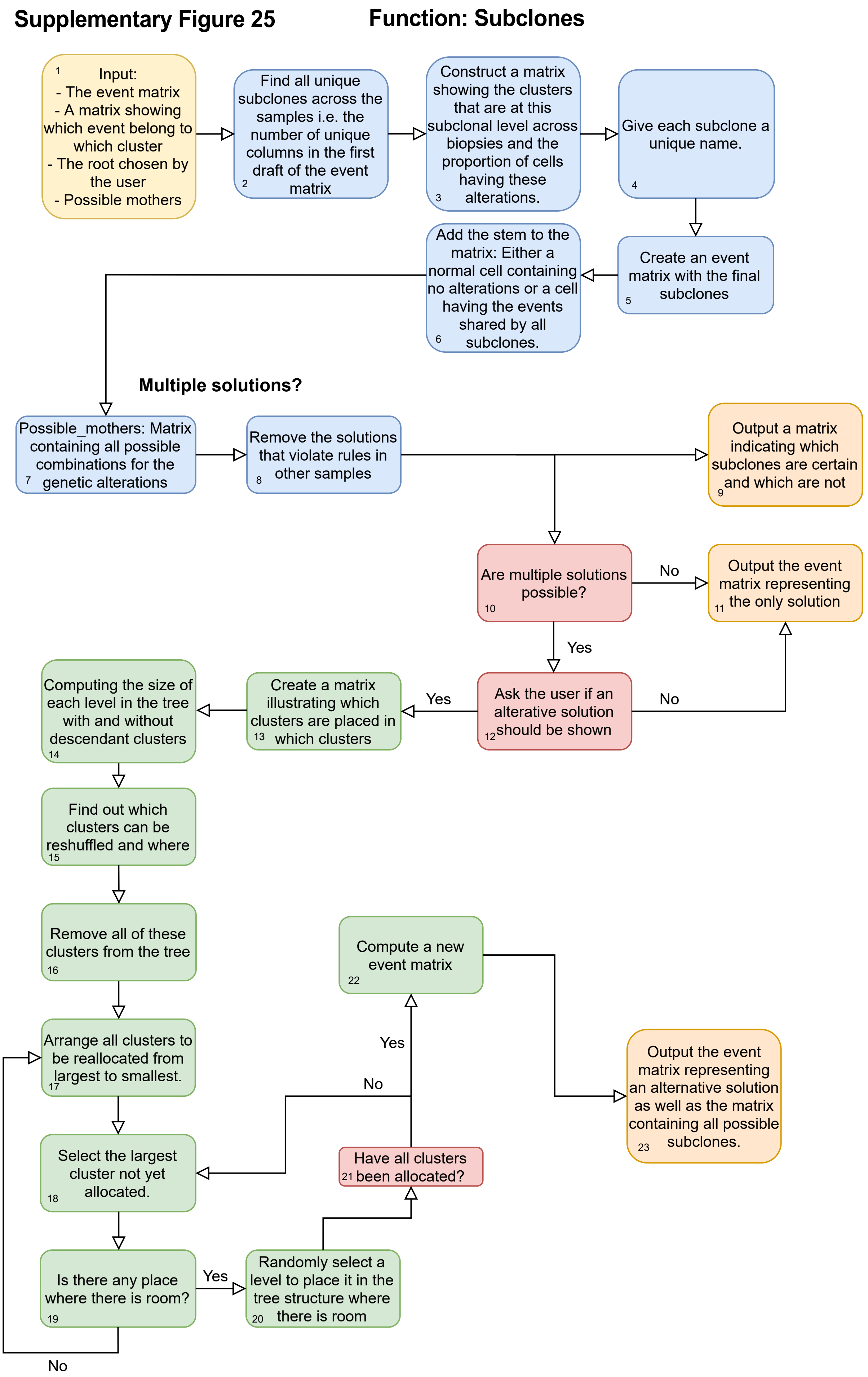

Supplementary Figure 25 Flow chart for the algorithm subclones. The input is the output file from the algorithm DEVOLUTION described in S.upplementary Figure 21-24. It extracts all unique subclones and their distribution across the biopsies. It also allows the user to choose if an alternative solution to the subclonal deconvolution should be shown. The output is a matrix indicating which subclones in the phylogeny are certain and uncertain as well as a final event matrix with all unique subclones across the samples.

Supplementary Figure 26 **Function: Make_pie**

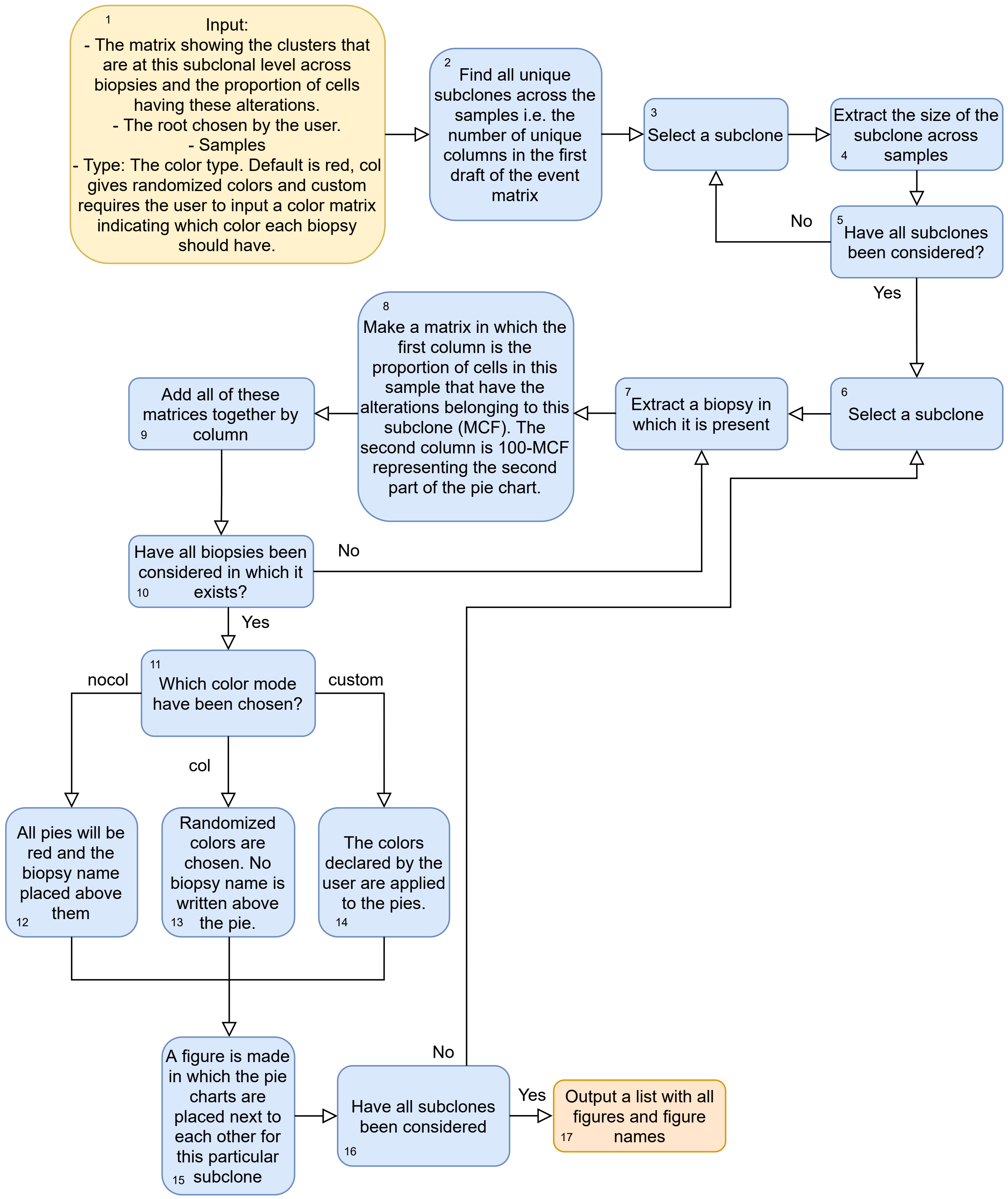

Supplementary Figure 26 Flowchart of the algorithm make_pie. Creating the pie charts based on how the subclones are distributed across samples.

Supplementary Figure 27 Function: Pie_it

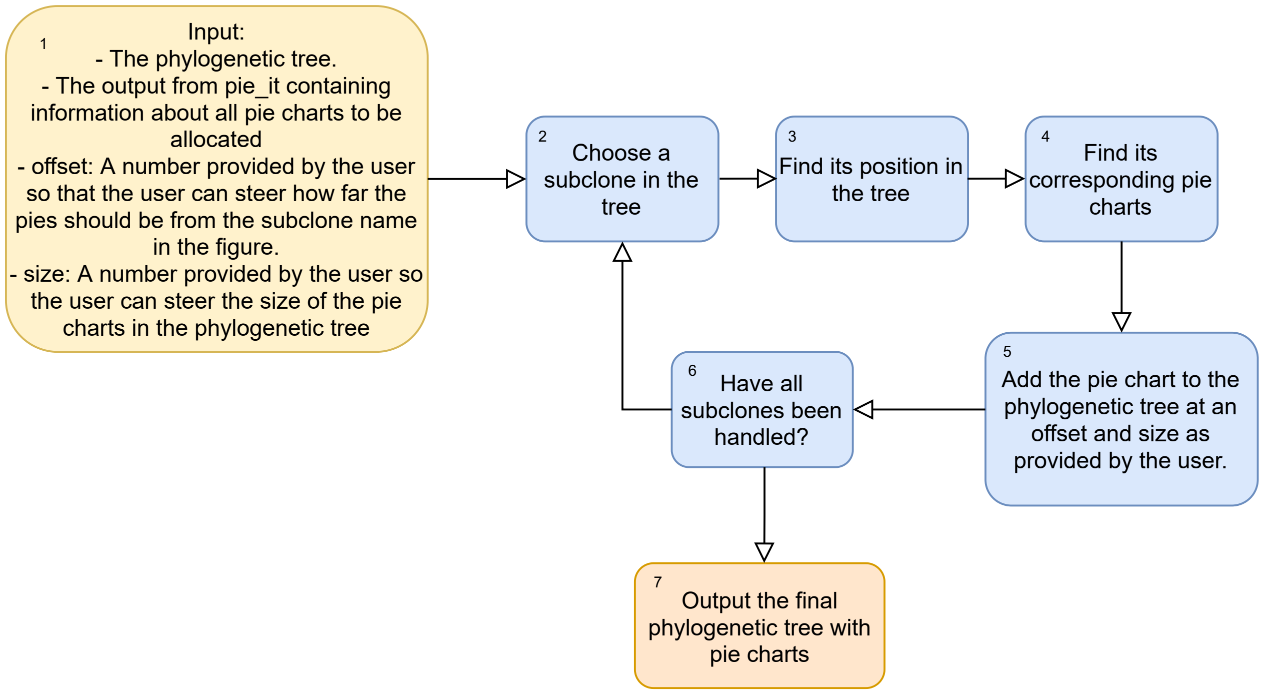

S.upplementary Figure 27 Flowchart for the algorithm pie_it. Creates the phylogenetic trees with pie charts.

Supplementary Figure 28 Function: phydatevent

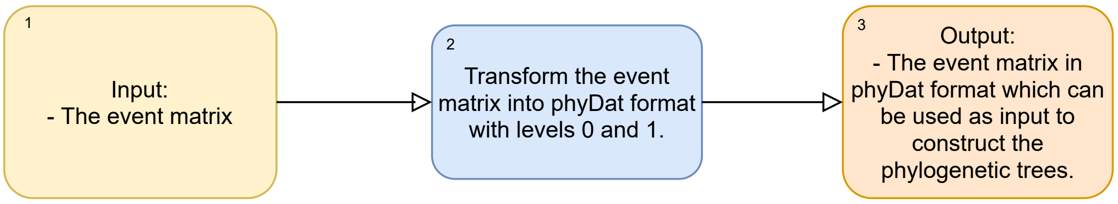

Supplementary Figure 28 Flowchart for the function phydatevent. Transforming the event matrix into phydat format in order for phylogenetic reconstruction using phangorn.

Supplementary Figure 29 DEVOLUTION.xlsx

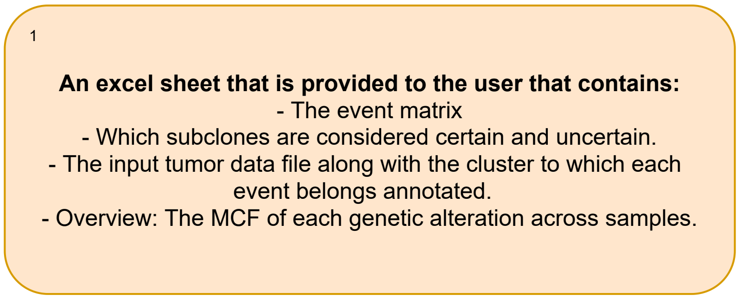

Supplementary Figure 29 The output excel file. Includes the event matrix, which subclones are considered certain and uncertain, the input data file together with the clustering as well as an overview matrix showing the MCF of each genetic alteration across samples.

Supplementary Figure 30 **RMS8**

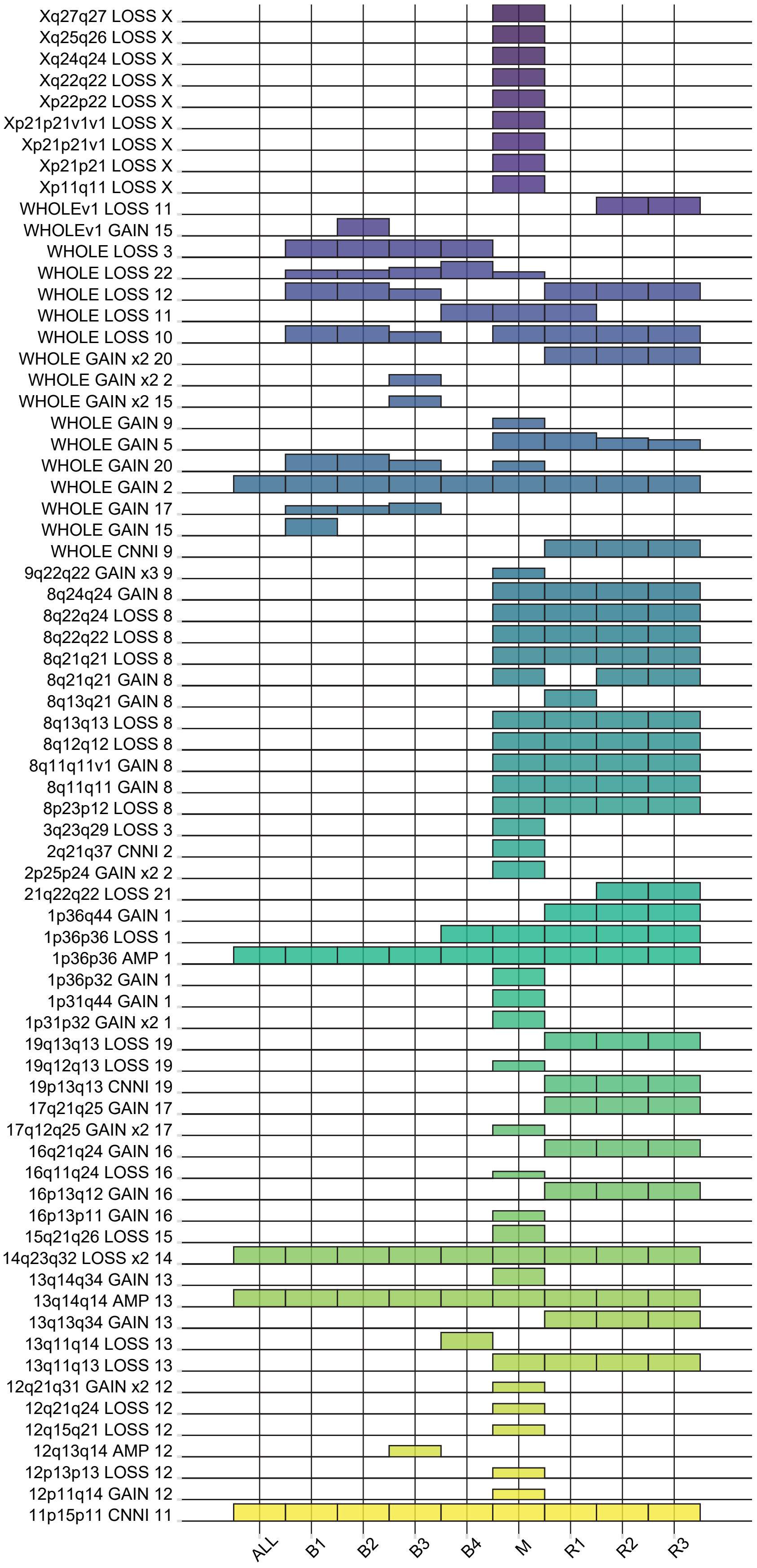

Supplementary Figure 30 A matrix visualizing the MCF for each genetic alteration across the biopsies for RMS8. Each row represents a genetic alteration and the columns the biopsies available for this patient. Each row is a histogram showing the MCF for a particular genetic alteration across samples. Here it is clear that some genetic alterations are present in all cells in all biopsies over time, hence constituting the stem events. The three biopsies constituting the local relapse R1-3 harbor an extensive repertoire of genetic alterations compared to the biopsies at diagnosis B1-3. They also have a clear resemblance to the metastasis, which harbors even more alterations. In addition, the biopsies from the local relapse share clonal genetic alterations not found in the metastasis, perhaps indicating that these cells might have disseminated early during the expansion of the relapse cells. This matrix of MCF-distributions for each genetic alteration across samples is what the DEVOLUTION algorithm uses to suggest the evolutionary

Supplementary Figure 31

Tumor ID	Sample ID	Chr	Start	End	Med LogR	VAF (TRS)	Type	Method	Cytoband/ Gene	Clone size (%)	
Tumor	ALL	3	63411	197852564	NA	NA	LOSS (1+0)	SNP array	WHOLE	100	ALL
Tumor	ALL	9	204738	140206472	NA	NA	LOSS (1+0)	SNP array	WHOLE	100	ALL
Tumor	ALL	16	83887	90158005	NA	NA	LOSS (1+0)	SNP array	WHOLE	100	ALL
Tumor	ALL	19	247232	59093239	NA	NA	LOSS (1+0)	SNP array	WHOLE	100	ALL
Tumor	ALL	21	9648315	48097610	NA	NA	LOSS (1+0)	SNP array	WHOLE	100	ALL
Tumor	B1	1	754192	46684266	-0.33	NA	LOSS (1+0)	SNP array	1p36p34	70	Cluster A
Tumor	B1	17	400959	38062217	-0.11	NA	GAIN (2+1)	SNP array	17p13q12	60	Cluster B
Tumor	B1	17	38074518	80263427	-0.11	NA	GAIN x2	SNP array	17q12q25	50	Cluster C
Tumor	B2	1	754192	46684266	-0.11	NA	LOSS (1+0)	SNP array	1p36p34	60	Cluster A
Tumor	B2	17	400959	38062217	0.3	NA	GAIN (2+1)	SNP array	17p13q12	50	Cluster B
Tumor	B3	2	25754005	27659491	0,3834862	NA	GAIN (2+1)	SNP array	2p23p23	90	Cluster D
Tumor	B3	5	38139	180698312	0,4063953	NA	GAIN (2+1)	SNP array	WHOLE	90	Cluster D
Tumor	B3	17	0	38299547	-0,13	NA	LOSS (1+0)	SNP array	17p12p11	50	Cluster E
Tumor	B3	17	0	38299547	-0,13	NA	GAIN (2+1)	SNP array	17p12p11	30	Cluster F

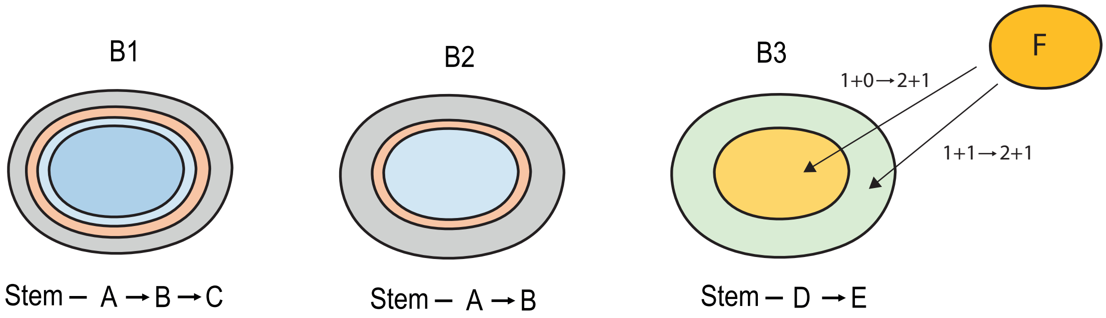

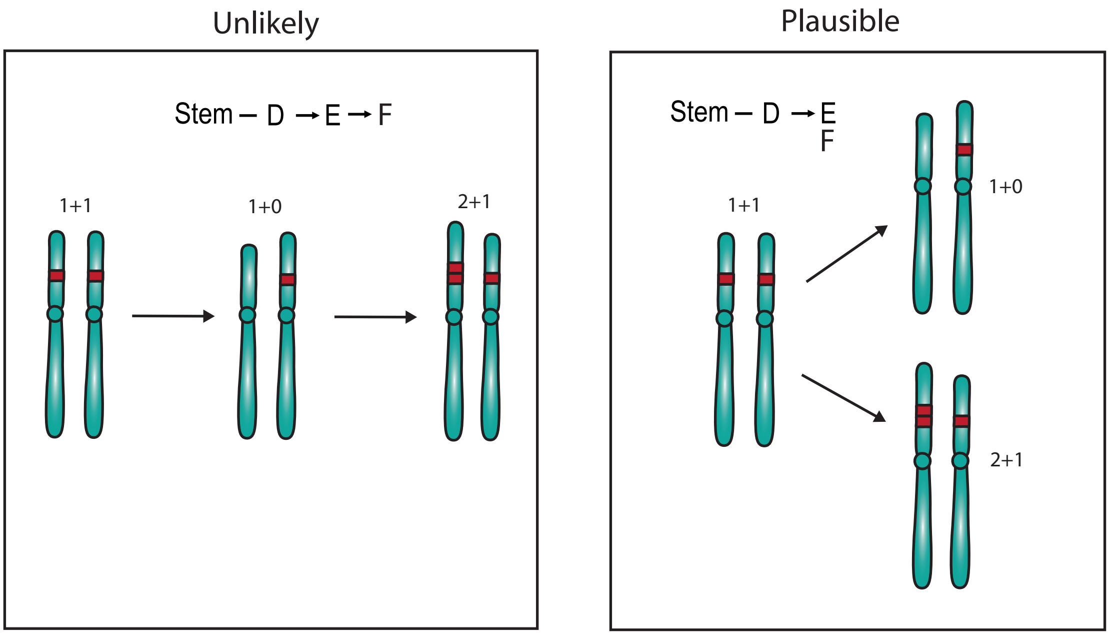

Supplementary Figure 31 Incorporating user-controlled rules for avoiding imposition of illicit biological trajectories. The table illustrates a fabricated tumor segment file with three biopsies, B1-B3. The ovals illustrate the suggested temporal order of the genetic alterations in this tumor. In biopsy B3 there are two different alterations of the same segment, hence a mixture of cells having different allelic compositions of the same chromosome segment. Cluster F could either be placed as a daughter after cluster E or directly after cluster D. Cluster E incorporates a loss of 17p12p11 to an allelic composition of 1+0, hence a loss of heterozygosity (LOH). An LOH can never become heterozygous again for that segment. Hence, the biological trajectory suggesting this is illicit. The user can supply the DEVOLUTION algorithm with a matrix specifying which genetic alterations in the data set cannot be placed after each other.

SUPPLEMENTARY METHODS

1.1 SUPPLEMENTARY NOTE 1 - THE PROPOSED IDEA

Using bulk genotyping provides information that can be used to compute the proportion of cells in each sample that have a particular aberration. To generate phylogenetic trees illustrating the relationship between subclones present in the tumor, which aberrations reside in the same cells as well as which subpopulations of cells the tumor consist of must be known. A subclone is defined as a cell having a unique genetic profile. By making use of a clustering algorithm, genetic alterations that follow similar patterns of mutated clone fractions (MCF:s) i.e. are present in the same proportion of cells, across biopsies can be identified. A true subclone of cells should produce a cluster of genetic alterations that persist. The alterations should remain grouped irrespective of inclusion of new data from an additional biopsied region of either the primary tumor or metastases. The goal of the clustering is to find the alterations that distinguish each subclone from its ancestor. To decipher the genetic profile of the subclones in the tumor, the clusters obtained through the clustering have to be nested, while taking into consideration information across samples. In this way, which genetic alterations reside in the same cells can be elucidated. See **Supplementary Figure 32** for an example.

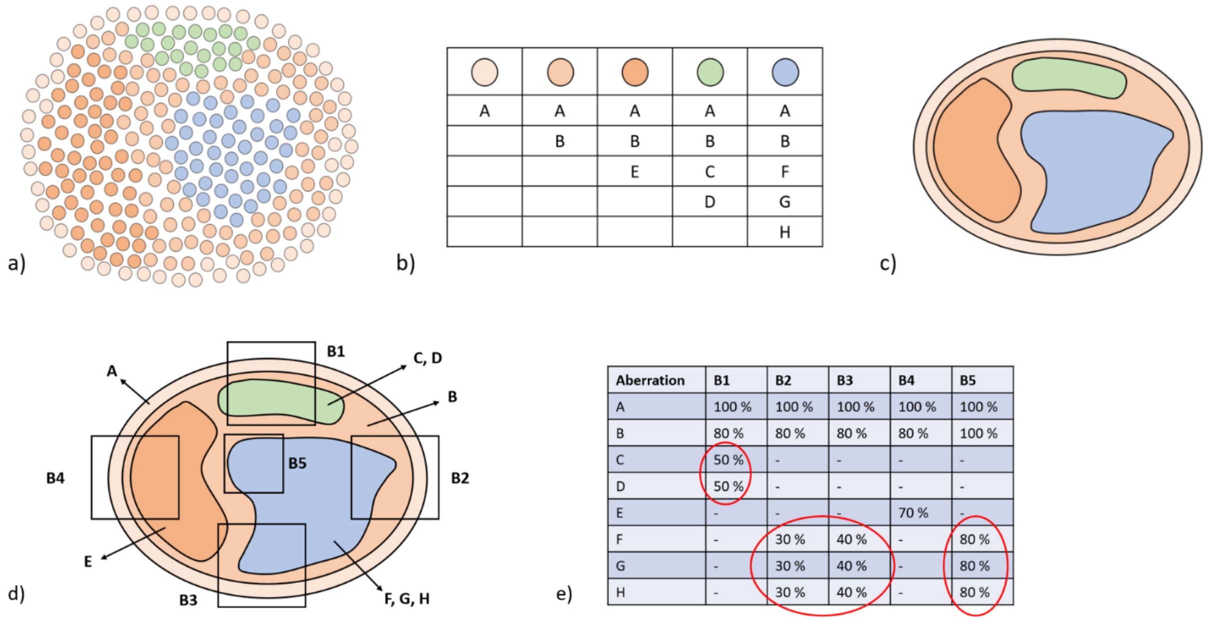

Supplementary Figure 32 The proposed idea a) A very simplified tumor consisting of cells harboring different sets of genetic alterations, indicated by different colors. **b)** A table illustrating the aberrations that the cells have. All of the cells have aberration A. The red light red cell in the second column have both mutation A and B and so on. **c)** Schematic illustration of the tumor in figure 1a. **d)** In a clinical situation some biopsies would be taken of the tumor. This is our window into the subclonal composition of the tumor. **e)** By searching for genetic aberrations that seems to follow each other over several biopsies the subclones can be reconstructed. In this example alteration A is in all cells and B in almost all cells. Alteration C and D are only found in the area comprised of biopsy B1. Alterations F, G and H seems to be in a subclone found in the area comprised of biopsy B2, B3 and B5 and alteration E is found in area B4. These former sets of aberrations are most likely in the same cells, while the latter aberration is not. The next step is to elucidate the temporal order of the events to obtain the subclones in the tumor. From the table we can identify groups of cells having alteration A, B, ABE, ABCD and ABFGH, which is in well accordance to b).

1.2 Supplementary note 2 - Structure

In order to analyze the data, an algorithm was created using the programming language R. The major structure of the program can be divided into five steps.

1. Preprocessing of the data.
2. Clustering of genetic alterations based on information from multi-region sampling from the same patient.
3. Subclonal deconvolution based on information from multiple samples from the same patient.
4. Construction of an event matrix.
5. Making use of a mathematical model to reconstruct the phylogenetic trees, in this case
    a. Maximum likelihood
    b. Maximum parsimony

1.3 Supplementary note 3 - Preprocessing of the data

The input data file should have the following format, exemplified here by WT11 (Wilms Tumor number 11) in Figure 2. Each row represents a genetic alteration in a biopsy specified by column 2. The method used to detect the genetic alteration is illustrated in column 9. Any method allowing the construction of a matrix as the one in **Supplementary Table 3** can be included in the analysis. Hence for example SNP-array, WES and WGS data can be combined in the same phylogeny.

1	2	3	4	5	6	7	8	9	10	11
Tumor ID	Sample ID	Chr	Start	End	Med LogR	VAF	Type	Method	Cytoband/ Gene	Clone size (%)
WT11	ALL	11	0	49669325	NA	NA	CNNI	SNP array	11p15p11	100
WT11	P1	7	0	56945077	-0,51	NA	LOSS	SNP array	7p22p11	60
WT11	P1	8	0	34592483	-0,49	NA	LOSS	SNP array	8p23p12	60
WT11	P2	7	0	56945077	-0,45	NA	LOSS	SNP array	7p22p11	50
WT11	P2	7	110628706	159119707	0,22	NA	GAIN	SNP array	7q31q36	30
WT11	P2	8	0	34592483	-0,44	NA	LOSS	SNP array	8p23p12	50
WT11	P2	8	47867326	146295771	0,22	NA	GAIN	SNP array	8q11q24	30
WT11	P3	7	0	56945077	-0,52	NA	LOSS	SNP array	7p22p11	60
WT11	P3	7	66433367	159119707	0,16	NA	GAIN	SNP array	7q11q36	25
WT11	P3	8	0	34592483	-0,50	NA	LOSS	SNP array	8p23p12	60
WT11	P3	8	35128512	146295771	0,17	NA	GAIN	SNP array	8q11q24	25
WT11	P4	7	0	56945077	-0,52	NA	LOSS	SNP array	7p22p11	60
WT11	P4	7	66433367	159119707	0,16	NA	GAIN	SNP array	7q11q36	25
WT11	P4	8	0	34592483	-0,51	NA	LOSS	SNP array	8p23p12	60
WT11	P4	8	35128512	146295771	0,17	NA	GAIN	SNP array	8q11q24	25
WT11	P5	7	0	56945077	-0,52	NA	LOSS	SNP array	7p22p11	60
WT11	P5	8	0	34592483	-0,52	NA	LOSS	SNP array	8p23p12	60
WT11	P5	8	35128512	41201081	-0,30	NA	LOSS	SNP array	8p11p11	40

Supplementary Table 3 A portion of the data obtained from SNP array analysis. In the first column the tumor ID is declared, in this case Wilms Tumor number 11. In the second column the biopsy name can be seen, indicating which aberrations have been found in which biopsy. Columns 3-5 shows the location of the alterations on the chromosomes. Columns 6 and 7 list the log2 median values for copy number aberrations and variant allele frequencies (VAF) for point mutations, respectively. Columns 8 and 10 represent the type of aberration. A GAIN means that there is one extra copy of this particular gene segment. Similarly, a LOSS indicates that one copy of the gene segment has been lost. The rightmost column gives the fraction of the cells in that particular biopsy that harbors this aberration, denoted the mutated clone fraction (MCF).

The input file containing the genetic alterations across biopsies, as in **Figure 2**, should be imported into the script from an excel sheet (xlsx) using the function **load_matrix** (**Supplementary Figure 19**). This makes sure it is in the correct configuration for downstream analyses.

The input file may contain information from multiple tumors i.e. matrices from different tumors may be positioned after one another in the sheet by the user. The function **splitdata** (**Supplementary Figure 20**) allows extraction of data from a particular tumor, which name is provided by the user. The user also obtains a matrix with the start and end positions (row numbers) for each tumor in the input file provided. It does not presuppose that the samples are sequentially arranged. This is done automatically by the algorithm splitdata. It arranges the rows by tumor, then by sample name within each tumor data set and subsequently from lowest to highest chromosome number within each sample.

1.4 SUPPLEMENTARY NOTE 4 - THE DEVOLUTION ALGORITHM

1.4.1 Preprocessing

When calling the function, the user can choose a **cutoff** for the genetic alterations in the segment file to be considered separate events, reflecting the measurement uncertainty of the start and end positions of the genetic alterations. The user also chooses whether the phylogenetic tree should be **rooted** in a normal cell (containing no alterations denoted "Normal") or a cell encompassing the alterations shared between the subclones (denoted "Stem"). Sometimes a mixed segment file with SNP array data, WES data etc. can be at hand. Therefore, the user can also choose **which data to include in the analysis**. In this way data from multiple different methods can be analyzed separately or in unison without having to separate the data manually.

Initially the algorithm checks if an event **cutoff** has been provided, if not a default of 1 Mbp is set (**Supplementary Figure 21 1-2**). Then it goes through the segment file to see if there are any **missing columns**. If there is a missing column a warning message is declared in the console. The missing column is replaced with "NA" (not a number) and the algorithm continues. If essential columns are missing the algorithm will halt (**Supplementary Figure 21 3-4**).

Subsequently **missing values** of MCF are identified. Missing values indicates that the MCF has not been able to be determined for a technical reason. This was changed to 100 % if the event did belong to stem, defined as the presence of the alteration in ≥ 90 % of the cells in all samples (default) or a cutoff provided by the user. The event was removed entirely if it was part of a subclone to not overestimate genetic variation within the tumor (**Supplementary Figure 21 5-6**). The user can also **declare which data to include** in the further analysis, for example to only include genetic alterations identified with SNP-array or WES. The user can declare it as "all" if all events, regardless of method, should be included. If not the "all" argument is given, alterations provided with a method not declared by the user, will be excluded from the data file for further analysis. In this way the user can in a simple way choose which combinations of methods to include for example to do one tree with SNP-array+WES, one with only SNP-array and one with only WES without having to rearrange the matrix by hand (**Supplementary Figure 21 7-8**).

1.4.2 Localizing unique genetic alterations

A clustering algorithm was constructed to localize all **unique genetic alterations** throughout the tumor samples. The algorithm loops through the rows of the segment file consisting of the genetic aberrations across the biopsies. For each row it compares the type of genetic alteration and their position on the chromosome to the other rows, representing other detected genetic aberrations throughout the samples. If two events in different samples are on the same chromosome, constitute the same type of alterations and if the events' start or end positions differ by less than a certain cutoff, set by the user based on the measurement uncertainty of the data set, they are considered the same type of event, but detected in different samples, otherwise they are considered different types of events in the evolution of the tumor. Each of these parameters are considered together in and and-statement. If they are identified as different events, a version name (i.e. v1, v2, v3 etc), is added to the alteration name for one of them, in order for the algorithm to be able to differentiate further on that they are different types of events (**Supplementary Figure 21 9-14**).

The default of the cutoff (co_{ev}) for DEVOLUTION is 1 Mbp (mega base pairs). Since the chromosome sizes ranges from 48-250 Mbp this cutoff constitutes a start and end point deviation of 0.4-2 % of the chromosome length.

All of the following conditions have to be met in order for the algorithm to consider the two alterations analyzed to be identical. See Figure 3 for an example.

1) *Alteration 1 and 2 are localized on the same chromosome.*
2) *Alteration 1 and 2 harbor the same type of alteration.*
3) *Neither alteration 1 nor 2 should belong to the stem.*

a. Alterations belonging to the stem are always considered separate events.

4) $X_1 = \|A_{1_{start}} - A_{2_{start}}\| \leq co_{ev}$

5) $X_2 = \|A_{1_{end}} - A_{2_{end}}\| \leq co_{ev}$

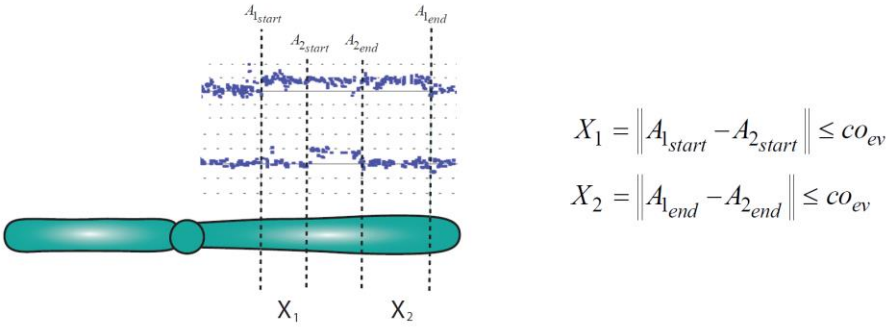

Supplementary Figure 33 Schematic representation of the comparison the algorithm makes. Alteration 1 and 2 have to be localized on the same chromosome, harbor the same type of alteration (for example GAIN, LOSS, CNNI etc) and neither should belong to the stem. Alterations belonging to the stem are always considered separate. The difference between the alterations' edges must be smaller than or equal to the cutoff chosen by the user reflecting the uncertainty in the measurement of the events.

The segment file was subsequently updated based on the clustering.

1.4.3 The overview matrix

An overview matrix was constructed, which is defined as a matrix that visualize the MCF of each genetic alteration across biopsies (**Supplementary Figure 21 15, Supplementary Figure 34**).

	ALL	B1	B2
17q11.2-q25.3 Gain (2+1)	100	100	100
17p11.2-p11.2 Gain (2+1)	100	100	100
22q11.23-q12.1 Loss (1+0)	100	100	100
3q13.31-q13.31 Loss (1+0)	0	60	80
19q12-q13.33 Loss (1+0)	0	30	80
8p23.3-p11.1 Gain (2+1)	0	10	20

Supplementary Figure 34 An example of an overview matrix. Each row represents a genetic alteration and each column the stem ("ALL") or a sample (here two biopsies, B1 and B2, are displayed). The numbers represent the MCF for each genetic alteration across samples.

A tumor is proposed to consist of multiple subpopulations of cells that harbor different sets of genetic alterations. Each individual alteration is part of a mutation space $m_i \in \{m_1, m_2 \dots m_\theta\}$ comprising all mutations present in the tumor where $i, \theta \in \mathbb{N}^+$ and θ is the total number of mutations. The mutational profile obtained from the biopsies thus represent a subset of the total mutation space and is the information at hand to describe the evolutionary trajectory of the tumor.

Let T_{MxB} be a matrix with the dimensions MxB which represents a particular tumor, where M is the total number of unique genetic alterations and B is the total number of biopsies. Hence m_δ indicates a certain genetic alteration δ, and b_ω represent a biopsy ω. The value $t_{\delta\omega}$ consequently corresponds to the MCF for an alteration, δ, in a sample, ω and $t_{\delta\omega} \in [0,100]$ i.e. it is bound between 0 and 100 %. This overview matrix can hence be written as

$$T_{MxB} = \begin{bmatrix} t_{1,1} & t_{1,2} & \cdots & t_{1,B} \\ t_{2,1} & t_{2,2} & \cdots & t_{2,B} \\ \vdots & \vdots & \ddots & \vdots \\ t_{M,1} & t_{M,2} & \cdots & t_{M,B} \end{bmatrix}$$

$$\text{where } t_{\delta\omega} \in [0,100], \delta \in \{1, \dots, M\}, \omega \in \{1, \dots, B\} \; and \; \delta, \omega \in \mathbb{N}^+$$

1.4.4 Stem algorithm

The next step is to see if there is a declared stem and if all stem events in the data set have been declared correctly. In this study the default definition of a stem is an event that is present in ≥ 90 % of the cells in all biopsies. The cutoff can be changed by the user. The algorithm loops through the overview matrix displaying the MCF:s across biopsies to identify which genetic alterations fulfill the criteria to be a stem event. The identified stem events are set to 100 % in all samples. They are subsequently removed from the segment file and added together in the beginning of the segment file. See an example below of the same segment file before and after passing through this algorithm. The overview matrix is updated with the changes and the stem events are placed in the beginning. This saves a lot of time for the user, since no manual stem declaration have to be made and it allows flexibility since the stem cutoff easily can be changed. If there for some reason is no stem event identified across the samples, the user will be notified through the console, and an artificial stem event will be added at the matrix header (**Supplementary Figure 21 16-22, Supplementary Figure 35**).

Before

Tumor	Sample ID	Chr	Start	End	Med.LogR	VAF..TRS.	Type	Method	Cytoband	Clone size
Tumor	B1	17	31621783	81041938	0,337806404	NA	Gain (2+1)	SNP-array	q11.2-q25.3	100
Tumor	B1	17	21545238	21865753	0,284071326	NA	Gain (2+1)	SNP-array	p11.2-p11.2	100
Tumor	B1	22	25656238	25922334	-0,59354758	NA	Loss (1+0)	SNP-array	q11.23-q12.1	100
Tumor	B1	3	116753539	116820186	-0,222	NA	Loss (1+0)	SNP-array	q13.31-q13.31	60
Tumor	B1	19	31110233	52680938	-0,36075759	NA	Loss (1+0)	SNP-array	q12-q13.33	30
Tumor	B1	8	158049	43837099	0,11869463	NA	Gain (2+1) P-arm	SNP-array	p23.3-p11.1	10
Tumor	B2	17	31621783	81041938	0,337806404	NA	Gain (2+1)	SNP-array	q11.2-q25.3	100
Tumor	B2	17	21545238	21865753	0,284071326	NA	Gain (2+1)	SNP-array	p11.2-p11.2	100
Tumor	B2	22	25656238	25922334	-0,59354758	NA	Loss (1+0)	SNP-array	q11.23-q12.1	100
Tumor	B2	3	116753539	116820186	-0,222	NA	Loss (1+0)	SNP-array	q13.31-q13.31	80
Tumor	B2	19	31110233	52680938	-0,36075759	NA	Loss (1+0)	SNP-array	q12-q13.33	80
Tumor	B2	8	158049	43837099	0,11869463	NA	Gain (2+1) P-arm	SNP-array	p23.3-p11.1	20

After

Tumor	Sample ID	Chr	Start	End	Med.LogR	VAF..TRS.	Type	Method	Cytoband	Clone size
Tumor	ALL	17	31621783	81041938	0,337806404	NA	Gain (2+1)	SNP-array	q11.2-q25.3	100
Tumor	ALL	17	21545238	21865753	0,284071326	NA	Gain (2+1)	SNP-array	p11.2-p11.2	100
Tumor	ALL	22	25656238	25922334	-0,59354758	NA	Loss (1+0)	SNP-array	q11.23-q12.1	100
Tumor	B1	3	116753539	116820186	-0,222	NA	Loss (1+0)	SNP-array	q13.31-q13.31	60
Tumor	B1	19	31110233	52680938	-0,36075759	NA	Loss (1+0)	SNP-array	q12-q13.33	30
Tumor	B1	8	158049	43837099	0,11869463	NA	Gain (2+1) P-arm	SNP-array	p23.3-p11.1	10
Tumor	B2	3	116753539	116820186	-0,222	NA	Loss (1+0)	SNP-array	q13.31-q13.31	80
Tumor	B2	19	31110233	52680938	-0,36075759	NA	Loss (1+0)	SNP-array	q12-q13.33	80
Tumor	B2	8	158049	43837099	0,11869463	NA	Gain (2+1) P-arm	SNP-array	p23.3-p11.1	20

Supplementary Figure 35 An example of how the input data file is changed when going through this part of the algorithm. In the top panel the original input data file is shown. No stem events have been declared by the user. In the bottom panel the algorithm has identified the genetic alterations across samples that fulfill the stem criteria. These alterations are consequently removed from the individual samples and aggregated at the head of the matrix.

1.4.5 Clustering genetic alterations

A clustering algorithm was then applied to group events that seem to have the same pattern over multiple samples. As discussed in the beginning of this document, alterations that follow each other in MCF over samples are most certainly within the same subclone in the tumor and is thus the group of genetic alterations that distinguish this group of cells from its ancestors. Note that these cells also can have other genetic aberrations, but they are not specific for this particular subgroup of cells compared to their ancestors. Simply identifying these clusters, is not the complete solution since nesting of the clusters still must be made.

In order to assess the similarity between the alterations from the different tumor regions, a clustering algorithm was used. Clustering in higher dimensions is difficult due to a divergence of the Euclidean distance between data points because they will deplete the center and concentrate in the shell of the n-dimensional space. In our case the dimensions consist of the number of samples, from the same patient, hence they are not completely independent of each other, and the total number of eigenvectors

is thus presumably lower than the total number of biopsies, which reduces the dimensionality and the problem.

Density based clustering techniques such as DBSCAN is efficient at clustering non uniform clusters and it allows clustering without specifying the number of clusters beforehand, which a pre-requisite with many other established clustering algorithms. In addition, it does only have two hyperparameters named *minPts*, which is the minimal number of points that is allowed in a cluster, and ϵ which is the radius in which points are included. The choice of ϵ can be aided by using a k-distance-graph which illustrates the distance to the minPts-1 = k nearest neighbor. The value to choose is when this plot shows an elbow (**Supplementary Figure 21 23 and 36**). The algorithm can be explained as follows,

1. *Randomly select a point p.*
2. *Retrieve all points that are density reachable from p with respect to ε and minPts.*
    a) *Density reachable: p* ϵ $N_{eps}(q)$ *and* $|N_{eps}(q)| \geq$ *minPts (core point) i.e. at least minPts have to be within a distance* ϵ*.*
3. *If p is a core point, a cluster is formed.*
    a) *Core point:* $|N_{eps}(q)| \geq$ *minPts*
4. *Continue the process until all points have been processed.*

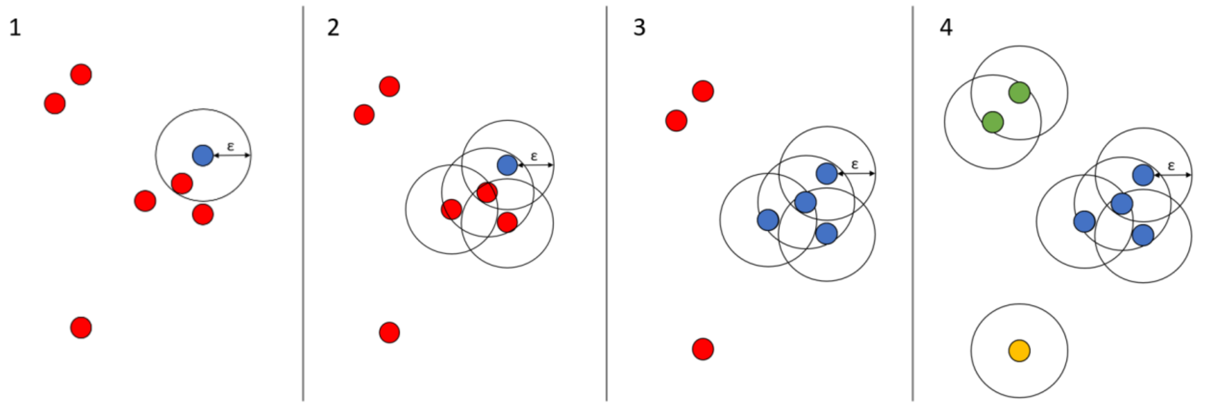

Supplementary Figure 36 Schematic illustration of the DBSCAN algorithm. 1) First a random point p is chosen. 2) Imagine a radius of length ϵ around the point p. We then find all points that are density reachable to the chosen point p. 3) If the obtained cluster size is at least minPts large, then a cluster is formed. 4) The process is continued until all points have been considered. The figure is adapted from the original paper for DBSCAN.

The clustering method can be changed in the code, and it is easy to add your own.

After the clustering, a matrix containing each cluster and its included genetic alterations is constructed. Let C_{KxN} be the matrix representing the clusters of genetic alterations. It has the dimensions KxN where k is the number of genetic alterations in the cluster and n is the cluster number. All matrix positions $C_{kxn} \neq 0$ are unique i.e. the same genetic alteration cannot belong to multiple clusters (**Supplementary Figure 21 24**).

$$C_{KxN} = \begin{bmatrix} c_{1,1} & c_{1,2} & \cdots & c_{1,N} \\ c_{2,1} & c_{2,2} & \cdots & c_{2,N} \\ \vdots & \vdots & \ddots & \vdots \\ c_{K,1} & c_{K,2} & \cdots & c_{KN} \end{bmatrix}$$

where $c_{kn} \in m_i \wedge c_{kn} \neq c_{ed}, (\forall\ k, e\ \in \{1, \dots, K\}\ \&\ n, d\ \in \{1, \dots, N\}\ \wedge c_{kn} \neq 0)$

The algorithm also constructs a matrix representing the clusters present in each biopsy and their size determined by the mean of the aberrations in the cluster (**Supplementary Figure 21 25**).

$$Z_{CxB} = \begin{bmatrix} z_{1,1} & z_{1,2} & \cdots & z_{1,B} \\ z_{2,1} & z_{2,2} & \cdots & z_{2,B} \\ \vdots & \vdots & \ddots & \vdots \\ z_{C,1} & z_{C,2} & \cdots & z_{CB} \end{bmatrix} \text{ where } z_{cb} \in [0,100]$$

Where c is a specific cluster of aberrations, b the biopsy and z_{cb} the size of the subclone in sample b.

1.4.6 Finding the possible allocations for each cluster

We now have the clusters of genetic alterations across biopsies. These clusters are although not the actual subclones, merely events that aid the identification of the subclones. The actual subclones will consist of a linear combination of these clusters of genetic alterations. To identify the subclones the clusters are nested, taking their prevalence in the biopsy in consideration and by combining information across biopsies in the process.

First, we need to define a **space matrix** including the subclonal partitioning of each biopsy. The space available in a single biopsy is 100 % and the space of all biopsies can thus be represented by a matrix where p is the partitioning of the available space in the biopsy and s_{pb} is the space available in a specific partitioning p in biopsy b. Initially $s_{1,b} = 100 \wedge s_{p\neq 1,b} = 0$ meaning that we start with the stem events in the bottom. The non-stem-clusters are then allocated to this space where there is still room for it to be placed. The space matrix can be represented as

$$S_{PxB} = \begin{bmatrix} s_{1,1} & s_{1,2} & \cdots & s_{1,B} \\ s_{2,1} & s_{2,2} & \cdots & s_{2,B} \\ \vdots & \vdots & \ddots & \vdots \\ s_{P,1} & s_{P,2} & \cdots & s_{PB} \end{bmatrix} \text{ where } s_{pb} \in [0,100] \text{ and } \sum_{p=1}^{P} s_{p,b} = 100 \ \wedge \ b \ \in \{1, \ldots, B\} \in \mathbb{N}^{+}$$

The clusters of aberrations in each biopsy, as supplied by Z_{CxB}, are allocated to the space in decreasing order, altering the magnitude of the spaces in S_{PxB} based on the MCF of the clusters allocated to it.

A biopsy is selected. The clusters identified in this biopsy are ordered from the one with highest MCF to lowest. In this way, there always will be at least one way to nest the clusters. The largest cluster not yet allocated is chosen. The space available in the biopsy is considered. The algorithm identifies in which clusters, already allocated, this cluster can be nested. The algorithm also considers if previously allocated clusters could be placed in another way to reveal additional possible allocation patterns.

The possible allocations for the cluster are catalogued in a matrix. A separate matrix, named **equalclones**, is introduced which includes cases where clusters of the same size (MCF) are nested in each other in order to keep track of these situations. The reason for this is that we do not yet know the order of the genetic alterations when the clusters are of the same size. To know that additional information from other samples is needed, which might reveal that one of the clusters have a smaller MCF than the other, providing information about the temporal order of these genetic alterations. When the information about the allocations have been saved, the largest cluster not yet allocated is extracted and the same procedure is repeated. This continues for all clusters in the biopsy and then it is repeated for all biopsies. In the end a matrix displaying the possible allocations for each cluster in each biopsy is obtained (**Supplementary Figure 21-22 26-36**). The next step will thus be to find a unified solution that is feasible across samples. Below (**Supplementary Figure 37**) is an example of this allocation process with the space matrix illustrated below.

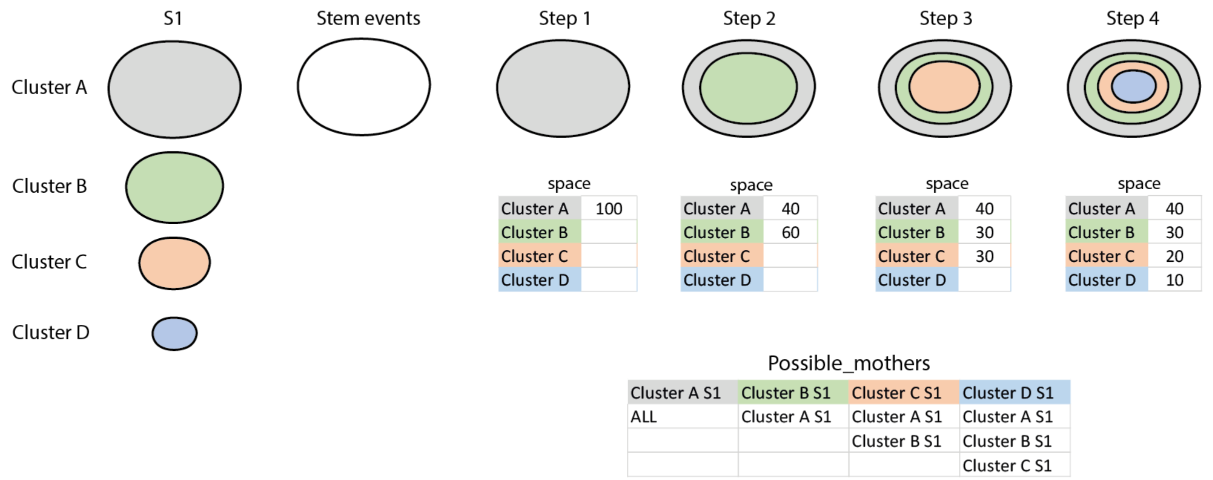

Figure 37 An example of the allocation of clusters. In this sample (S1) there are 4 identified clusters of genetic alterations, cluster A, B, C and D. The genetic alterations in these clusters have an MCF of 100 %, 60 %, 30 % and 10 % in S1 respectively. As the clusters are allocated to the space the space matrix visualized below the ovals change. Initially the space is merely occupied by the stem events. In the first step there is only one possible allocation of Cluster A S1 (denotation of cluster A in sample 1). This cluster then takes over the entire space of the matrix, meaning that additional genetic alterations in the biopsy must be equal or later in the temporal evolution of the tumor. In the next step cluster B S1 is nested in Cluster A S1 since this is the only position for nesting feasible. Cluster C S1 can be nested both in Cluster A S1, resulting in the presence of subclones of cells that have the genetic profile of cluster A + C, or nesting in cluster B giving cells having cluster A+B+C. Cluster D S1 can be nested at all levels. If the clusters are nested consecutively, as in the top row of ovals, the space will change as is visualized by the matrix below them. In possible_mothers the possible allocations for each cluster are saved.

1.4.7 Looking for discrepancies between samples

As was discussed in the previous section, there might be multiple solutions. If there are multiple biopsies available, information can be combined from each biopsy to find a unified solution not in disagreement with any of the samples.

Before doing this, the equalclones matrix is revisited to make sure that the equalclones actually can be placed together by analyzing their pattern across samples. They are not allowed to *both* be **prevalent without one another**. They are not allowed to **cross each other in MCF** across biopsies. There may also be cases in which it is **not even possible to nest them together** in some samples even though they both are prevalent (**Supplementary Figure 22 37, Supplementary Figure 38**).

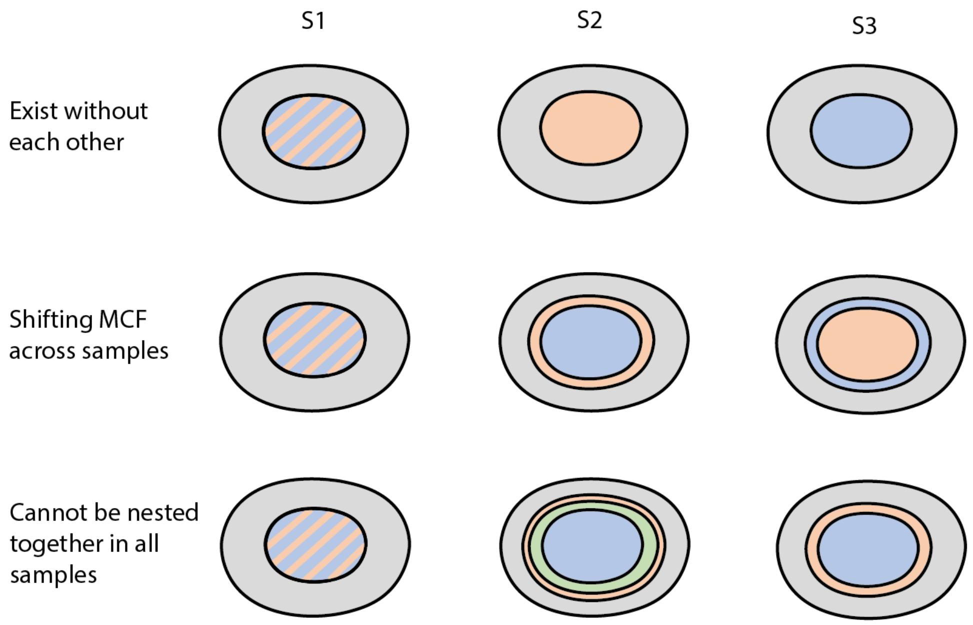

Supplementary Figure 38 Illustration of how information across samples (here S1, S2 and S3) can be unified to conclude that two clusters cannot be nested. Three different scenarios are displayed to exemplify this. In the first sample, S1, two clusters are nested together and consequently also added to the equalclones-matrix. In the top row the clusters are found without one another in separate samples. Hence there exist cells having the alterations of these clusters independently, making it unlikely that these alterations are in the same cells. In the middle row the MCF values cross one another across samples. In the bottom row nesting if not possible in all samples, even though they are both present. In S2 the blue cluster must be nested in the green cluster which in turn is nested in the red cluster, while the blue cluster is nested in the red in S1 and S2. Hence the temporal evolution of the genetic alterations across samples are contradictory for this solution and the most probable solution is that these clusters are present in separate cells and should not be nested in S1.

Subsequently the equalclones should get each other's mothers, as well as each other as mother, in possible_mothers in the samples in which they are of equal size (**Supplementary Figure 22 38, Supplementary Figure 39**).

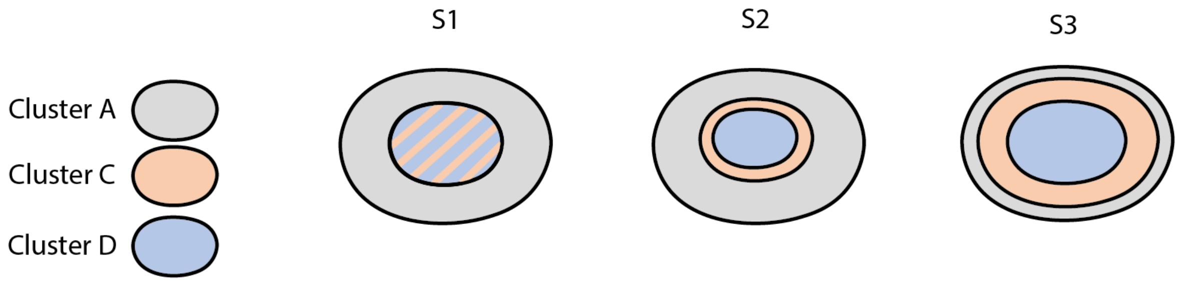

Possible_mothers

Cluster A S1	Cluster C S1	Cluster D S1	Cluster A S2	Cluster C S2	Cluster D S2	Cluster A S3	Cluster C S3	Cluster D S3
ALL	Cluster A S1	Cluster A S1	ALL	Cluster A S2	Cluster A S2	ALL	Cluster A S3	Cluster C S1
	Cluster D S1	Cluster C S1			Cluster C S2			

Supplementary Figure 39 Clusters of the same size that are nested in a particular sample should get each other's mothers in that sample. In sample S1 cluster C S1 and cluster D S1 are nested in each other. Solely based on this sample it cannot be determined if a cell have gotten the cells of cluster A, followed by C and then D or if the cells have obtained A first, followed by D and then C. Hence, the clusters get each other's mothers when they are nested in a sample and are of the same size. By including information from the other samples, it can be determined that cluster D should be nested in C which in turn is nested in A, which stresses the need to render the possible_mother matrix in subsequent steps to obtain the most probable solution of the temporal order of the clusters.

The user is also allowed to provide rules for the nesting of clusters of genetic alterations. Some genetic alterations may be known through studies, for example, to never co-occur. In those cases, the user can

provide the algorithm with a rule, forbidding these events to be placed in the same cell. This rule is taken as input by the DEVOLUTION algorithm in the shape of a matrix with two columns in which events in the same row are the ones that should not be nested in each other (**Supplementary Figure 22 39-40**).

The next step is to look for a unifying solution between the samples of the tumor. We want to make an allocation of the clusters that does not contradict any of the biopsies. We fuse the information from multiple biopsies to extract the trajectory of genetic alterations that is most probable. If the data set only contain information from a single biopsy, no such comparison is needed or possible, but the algorithm can still do the nesting and produce a phylogeny for the user. There is always a unique solution. If there are multiple biopsies each cluster is initially considered separately.

First, a cluster not yet processed is chosen. The possible nesting of this cluster across the biopsies in which it exists are extracted. A table is constructed that shows in how many of the samples each nesting is possible. Hence identifying which nestings is possible in all, or almost all samples, and which nestings are only possible in a subset of biopsies, making this a less probable nesting pattern. The clusters that can be selected as the mother cluster in all biopsies are identified. Ideally there would only be **one single possible allocation** that is feasible in all samples. If there is only one, that is chosen as the mother cluster. Sometimes there may be **multiple solutions equally probable**. In that case we first assess if the user has provided the algorithm with a rule forbidding certain events to be placed together in the same cells. If so, those allocations are removed. As a next step the MCF of each cluster across samples is extracted and **how the MCF-sizes changes across the samples** are assessed. Mothers that rise and fall in relation to the daughter cluster are selected as potential mother candidates. If there are multiple such, the one providing the largest space is chosen, unless there is a rule provided by the user, in which case that rule overrides the solution. If there is one single cluster that follows the daughter cluster in size across biopsies, that cluster is chosen. If no cluster follow the daughter clone in sizes and no rules aid us in the choice of allocation, the largest space is chosen. In the end of this part of the subalgorithm whether any of the mothers are not possible any more due to previous allocations of clusters. There may for example be some other cluster that have to be nested in this mother cluster, which decreases the space available, which might lead to the cluster handled now to not be able to be allocated to that position anymore. In that case, it is removed as a possible_mother for the daughter cluster. The other possible mothers are moved accordingly in the matrix, where the first row represents the cluster that is the preferred mother cluster and the other clusters less preferred, given the rules above. Hence, if the preferred solution is removed through this algorithm, the second most preferred allocation is chosen instead (**Supplementary Figure 40**).

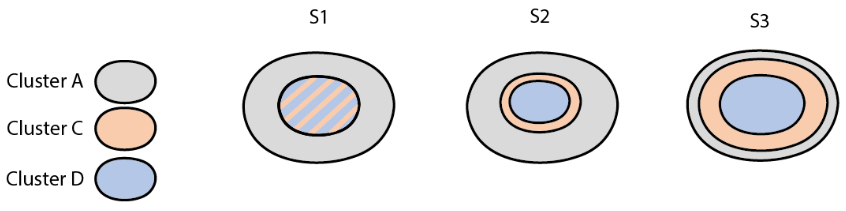

Cluster A	
ALL	3
**Cluster C**	
Cluster A	3
Cluster D	1
**Cluster D**	
Cluster A	2
Cluster C	3

Possible_mothers

Cluster A S1	Cluster C S1	Cluster D S1	Cluster A S2	Cluster C S2	Cluster D S2	Cluster A S3	Cluster C S3	Cluster D S3
ALL	Cluster A S1	Cluster A S1	ALL	Cluster A S2	Cluster A S2	ALL	Cluster A S3	Cluster C S1
	Cluster D S1	Cluster C S1			Cluster C S2			

Supplementary Figure 40 An example of the table constructed that shows in how many of the samples each nesting is possible. Cluster A can be placed after the stem events in all samples. No other nestings are possible for this cluster. Cluster C can be nested in D in one sample (in which they are equalclones). In the other two samples it cannot be nested into cluster C as a daughter cluster. It can although be nested in cluster A in all three samples, making this the most probable allocation. Cluster D can be nested in A in two samples. In the third sample it cannot. It can be nested in cluster C in all biopsies. The temporal order of the clusters will thus be: the stem → Cluster A → Cluster C → Cluster D. In this case there is only one nesting for each cluster that is possible in all samples. Hence there is only one solution that does not give rise to parallel evolution or back mutations.

There might be a situation where there is **no allocation** of the cluster that is allowed in all samples. We first assess whether some allocations are the only solution in any samples or there are rules provided by the user, for which those clusters are given. If there are no rules, we look for allocations possible in all but one sample. When there is no indication of which is the correct allocation the one providing the largest space is chosen.

The procedure is repeated for all clusters identified across the biopsies. We now have a determined allocation for every cluster across the biopsies. To minimize the risk for contradictions in the allocations due to allocations of previous clusters, the subalgorithm is rerun once to make sure all mothers are still possible when they are allocated. This takes minimal extra time for the code (< 0.2 sek) but provides a more robust output (**Supplementary Figure 22 41-71**).

1.4.8 Algorithm for making sure clusters are allocated in the correct order in all samples

The aim of this algorithm is to nest the clusters across the biopsies in the order determined by the algorithm described in the previous section. In the process the final partitioning of the tumor space i.e. the spaces left at each level of the nesting, corresponding to the proportion of cells in the biopsy belonging to a certain subclone is computed as well.

A biopsy is selected followed by extraction of all clusters identified within that biopsy and their corresponding MCF:s. These clusters are the ordered from largest to smallest. By doing this we make sure there will always be at least one solution for the nesting of each biopsy. The largest cluster not yet considered is chosen. Initially we start with a space in which 100 % of the cells have the genetic alterations i.e. they have the stem event. Hence there will only be one single space, one of the size 100 for the new cluster to be placed in. In general, the largest space available is always identified in the beginning of the algorithm.

If **there is a rule** for the allocation of this cluster, either provided by the user, it is the single solution in the sample or that a particular allocation has been specifically chosen in the algorithm above, we extract the mother cluster it must be nested in. We double check that this mother even exists in the sample and that there is enough room for the daughter cluster to be nested in it. If this is not the case, the second most preferred mother in possible_mothers is chosen. If not present the largest space is chosen. We also save information in a matrix for the cases in which the cluster could not be allocated correctly. Another case may be that the mother exists in the sample but has not been allocated yet. This mother cluster (eg. M1) may also have a mother cluster (eg. M2) that have not been allocated yet, in which case the clusters are allocated such that M2 is allocated to the space first, then M1 is nested within that and they the daughter cluster is nested in M1. If M2 has been allocated we nest M1 in M2 and then the daughter cluster in M1. If there is no rule for where the mother should be placed, it is allocated to the largest space. If **there is no rule** for the allocation of a certain cluster the allocation the allocation determined by the algorithm where we considered discrepancies between samples is used.

In the next step whether the mother or daughter cluster is in equalclones is considered. For example, if a cluster A consist of 5 genetic alterations and have an MCF of 80 % and another cluster B have 3 genetic alteration that also have an MCF of 80 % in this particular sample, these clusters will be nested. This means that 80 % of the cells in this biopsy will have both the genetic alterations of cluster A and B. If another cluster C is 40 % the cells encompassing these 40 % of the biopsy will have the genetic alterations of cluster A, B and C. In another sample the MCF:s might differ from one another such that we know that cluster A > B > C. Hence C will be nested in B and B nested in A. This order of the nesting must be taken into account when constructing the event matrix.

There are four different situations that can occur:

- **The mother is in equalclones but not the daughter:** Then we will remove the daughter's space from all clusters in the equalclones including the mother cluster. If the daughter and

mother are equal in size, the daughter should be included in equalclones together with the mother.

- **The mother and the daughter are in equalclones:** If they are of the same size no spaces are removed. The clusters are simply nested together. Double check if they are placed in the same column in the equalclones columns for this sample. Otherwise, it should be moved such that it is. If the mother and daughter cluster are not of the same size, the size of the daughter is subtracted from the mother's space along with the equalclones placed together with it.
- **The mother is not in equalclones but the daughter is:** If they are of the same size no spaces are removed. The clusters are simply nested together. We must make sure the mother cluster is added to equalclones together with the daughter.
- **None of them are in equalclones:** If they are of the same size they should both be added to equalclones. No spaces are removed. If they are not of the same size the daughter's space is removed from the mother's.

The mother cluster chosen for each daughter cluster is saved in allocation_samples. The process is repeated until all clusters have been processed in all biopsies. In the end we will have a complete allocation_samples matrix that shows the nesting of all clusters across biopsies (**Supplementary Figure 22-24 72-106**).

1.4.9 Creating the event matrix

So, now the nesting of all clusters of genetic alterations across the biopsies is known. The next step is to construct the event matrix based on this nesting. See Figure 9 for an example.

An algorithm was constructed that controls the validity of the final equalclones matrix. There may be clusters in this matrix that in the end got nested at a different position, in which case it should be removed from the matrix (**Supplementary Figure 24 107**).

In this first version of the event matrix, each row represents a genetic alteration and each column the identified subclones across samples. At this stage, the same subclone can appear multiple times, but in different samples. This gives us information about its size across samples and in which samples it is found and not.

An event in the event matrix is chosen, followed by a biopsy and then a cluster within that biopsy. If the event is present in this cluster a "1" is added in the event matrix at the column representing this cluster in this biopsy. Repeat this procedure for all genetic alterations. In the end the matrix will show the events encompassed in each cluster. Then a "1" is added in all columns for the events incorporated in the stem, which should be present in all cells (**Supplementary Figure 24 108-116**). The next step is to make sure the determined nesting pattern is correctly translated into an event matrix.

A biopsy is selected followed by a daughter cluster within in that sample. The mother cluster it should be nested into is identified using the **allocation_samples-matrix** in which the nesting pattern is displayed. A matrix is constructed in which the first column is the daughter cluster, the second the mother cluster and the third the MCF of the mother cluster. The matrix is then ordered from largest to smallest. Choose the largest one not yet considered. If the daughter is part of equalclones the other clusters within this should also have the events of this mother. If it is not part of equalclones the daughter is simply given the events of the mother in the event matrix. This is repeated until all clusters in all samples have been handled. Then the output event matrix is obtained (**Supplementary Figure 24 117-131, Supplementary Figure 41**).

	Cluster A S1	Cluster C S1	Cluster D S1	Cluster A S2	Cluster C S2	Cluster D S2	Cluster A S3	Cluster C S3	Cluster D S3
Mut 1	1	1	1	1	1	1	1	1	1
Mut 2									
Mut 3									
Mut 4									
Mut 5									
Mut 6									
Mut 7									
Mut 8									
Mut 9									
Mut 10									

All subclones should have the events of the stem

	Cluster A S1	Cluster C S1	Cluster D S1	Cluster A S2	Cluster C S2	Cluster D S2	Cluster A S3	Cluster C S3	Cluster D S3
Mut 1	1	1	1	1	1	1	1	1	1
Mut 2	1			1			1		
Mut 3	1			1			1		
Mut 4	1			1			1		
Mut 5		1			1			1	
Mut 6		1			1			1	
Mut 7		1			1			1	
Mut 8		1			1			1	
Mut 9			1			1			1
Mut 10			1			1			1

Give each column its cluster's correspondin events.

	Cluster A S1	Cluster C S1	Cluster D S1	Cluster A S2	Cluster C S2	Cluster D S2	Cluster A S3	Cluster C S3	Cluster D S3
Mut 1	1	1	1	1	1	1	1	1	1
Mut 2	1	1	1	1	1	1	1	1	1
Mut 3	1	1	1	1	1	1	1	1	1
Mut 4	1	1	1	1	1	1	1	1	1
Mut 5		1	1		1	1		1	1
Mut 6		1	1		1	1		1	1
Mut 7		1	1		1	1		1	1
Mut 8		1	1		1	1		1	1
Mut 9			1			1			1
Mut 10			1			1			1

Give each cluster the events of its mothe

	Cluster A S1	Cluster C S1	Cluster D S1	Cluster A S2	Cluster C S2	Cluster D S2	Cluster A S3	Cluster C S3	Cluster D S3
Mut 1	1	1	1	1	1	1	1	1	1
Mut 2	1	1	1	1	1	1	1	1	1
Mut 3	1	1	1	1	1	1	1	1	1
Mut 4	1	1	1	1	1	1	1	1	1
Mut 5		1	1		1	1		1	1
Mut 6		1	1		1	1		1	1
Mut 7		1	1		1	1		1	1
Mut 8		1	1		1	1		1	1
Mut 9		1	1			1			1
Mut 10		1	1			1			1

Take equalclones into consideration.

	Subclone A	Subclone B	Subclone C
Mut 1	1	1	1
Mut 2	1	1	1
Mut 3	1	1	1
Mut 4	1	1	1
Mut 5		1	1
Mut 6		1	1
Mut 7		1	1
Mut 8		1	1
Mut 9			1
Mut 10			1

The final event matrix

Three different cells have been identified across the biopsies.

Supplementary Figure 41 An overview of the translation of the nesting pattern into an event matrix in order to identify the subclones across samples. Each one should have the events of the stem. Then the events corresponding to each cluster is allocated to the corresponding column. As a next step the temporal order or nesting of the clusters and equalclones are taken into consideration. This results in an event matrix containing the subclones in each sample. Using the function Subclones, described in the next section, a matrix is produced that contains the unique subclones along with information about their corresponding sizes across samples.

1.4.10 Function: Subclones

This function identifies all subclones across samples, defined as a cell population having a unique genetic profile. The algorithm also identifies additional possible solutions for the phylogenetic tree structure, which the user can assess to produce alternative phylogenetic trees that still explain the data set at hand.

First, all unique subclones across the samples are identified. This is done by finding all unique combinations of the genetic alterations in the event matrix and giving them new unique subclone names, creating a new event matrix in which each column is unique. Also, a column representing the stem is added. This is either a normal cell or a cell containing all alterations fulfilling the criterion for being a stem event (**Supplementary Figure 25 1-6, Figure 41 bottommost panel**).

By making use of the phylogeny and possible_mothers a matrix showing which clusters are placed at each level in the phylogeny across biopsies as well as the proportion of cells having these alterations is constructed. Hence for each level corresponding to different subclone names in the phylogeny, which clusters are nested on top of this as well as the size of these clusters are known (**Supplementary Figure 25 3**). Clusters violating rules in other samples are removed. At this step, a matrix is given to the user containing which subclones in the phylogeny are certain (there is only one possible allocation of the clusters resulting in this subclone) or uncertain (the clusters contained in this subclone could be allocated to other places such that this subclone does not exist) (**Supplementary Figure 25 7-9**).

If at least one subclone is uncertain there are multiple solutions for the phylogeny. In that case, the user is asked whether an alternative solution should be shown or not. If the user does not want that, the suggested solution is shown. If the user would like to opt for an alternative solution, as a first step a matrix is created that illustrates which clusters across biopsies are nested in which. In this matrix there cannot be any duplicates. For example, cluster A in a biopsy 1 (denoted B1_cluster_A) in Figure 7 can only be nested in one single position. Based on this information the size of the space in each level of the tree with and without the nested clusters can be computed (**Supplementary Figure 25 10-14, Supplementary Figure 41**).

The user can choose a cutoff for which subclones to reshuffle. The matrix showing certain and uncertain subclones can be used as an aid for selection of this cutoff. The default is 30 %. In that case, all clusters < 30 % in all samples will be randomly reshuffled in the tree. To do this all clusters fulfilling this requirement are removed from the phylogeny, leaving a tree only containing the subclones for which there is only one nesting pattern of the encompassed clusters. The disconnected clusters are arranged from largest to smallest. The largest cluster not yet allocated is chosen. The algorithm finds at which levels of the tree i.e. after which subclones this cluster could be allocated, without breaking any of the previously established rules for the allocation of this cluster. One of the allocations fulfilling this is randomly chosen. This alters the spaces remaining in the tree. Then the next disconnected cluster not yet considered is chosen and the process repeated. When all clusters have been reestablished in the phylogeny an alternative solution is at hand. The user will get a new event matrix as an output (**Supplementary Figure 25 15-24**) for which a phylogeny can be reconstructed.

The complete number of alternative solutions can be very large for cases in which a vast number of clusters with small MCF:s are identified across samples. The phylogenetic tree structure may still not change as much. There may be a couple of clusters with large MCF:s that only can be nested in a single way and then a lot of small clusters of very small MCF:s that can be nested in many combinations, both within the large clusters and within each other, resulting in a large quantity of phylogenies, but where the main structure of the tree remains stable. Hence, the number of possible phylogenies, provides very little information about the reliability of the phylogeny at hand. The user can, through the algorithm, choose whether or not to color the tip labels for the subclones depending

on which ones were created using clusters for which a single solution was possible and for which multiple solutions were possible, providing a visual cue of stable and dubious parts of the phylogeny.

1.5 Supplementary note 5 - Function: make_pie

This functions loops through all subclones and extracts its size across samples. Then it produces a matrix in which the first column is the proportion of cells in this sample that have the alterations belonging to this subclone and the second column is this size minus 100. Together these values across biopsies are used to produce the pie charts belonging to each subclone (**Supplementary Figure 26 1-10**).

The user can choose between three different color modes for the pie charts.

- **Nocol:** The pie charts have the default color red. Above the pie charts their corresponding sample name is illustrated.
- **Col:** For each sample, a color is randomly picked to represent it. The pie charts will hence be colored and there will not be a sample name above the pie charts. A legend is produced that indicated which color corresponds to which sample.
- **Custom:** The user can itself choose which color each sample should be colored with.

The output is one figure for each subclone in which the pie charts are arranged besides one another (**Supplementary Figure 26 11-17**).

1.6 Supplementary note 6 - Function: pie_it

It adds the pie charts to the phylogenetic trees. As an input the user have to provide the phylogenetic tree, the pie charts, a number for the offset of the pie charts relative to the subclone names and a number for the size of the pie charts. The offset and size may have to be altered based on the size of the phylogenetic tree.

The algorithm loops through the subclones in the tree. It finds its position, its corresponding pie chart and adds the pie chart to the corresponding position (**Supplementary Figure 27 1-7**).

1.7 Supplementary note 7 - Function: phydatevent

Transforms the event matrix into phyDat format so that it is compatible with the phangorn package to produce the trees (**Supplementary Figure 28 1-3**).

1.8 Supplementary note 8 - Excel sheet output

From the algorithm there will be an excel sheet provided that includes the event matrix, which subclones in the phylogenetic trees are considered certain and uncertain, the segment file with the cluster to which each event belongs annotated as well as an overview matrix containing the MCF of each genetic alteration across samples (**Supplementary Figure 29 1**).
