# Supplementary material for: DEVOLUTION—A method for phylogenetic reconstruction of aneuploid cancers based on multiregional genotyping data
Source: Commun Biol. 2021 Sep 20;4:1103. doi: 10.1038/s42003-021-02637-6 (PMC8452746; doi:10.1038/s42003-021-02637-6)
Supplement: Supplementary file 3 — Description of Additional Supplementary Files [file 42003_2021_2637_MOESM3_ESM.pdf]

Description of Additional Supplementary Files

File name: Supplementary Data 1.
Description: All segment files for NB, WT and RMS along with the clustering obtained by the software. Segments are annotated similar to Supplementary Figure 1.

File name: Supplementary Data 2.
Description: The final event matrices.

File name: Supplementary Data 3.
Description: The segment file for each simulation, the biopsy positions randomly chosen, which unique cells are present in these biopsies, the event matrix and evaluation of which alterations are correctly allocated using the software.

File name: Supplementary Data 4.
Description: Tables illustrating the number of alterations in total, alterations with a clone size > 10 % and the number of alterations correctly allocated.

File name: Supplementary Data 5.
Description: This table includes segment files and corresponding event matrices on which the phylogenetic trees are based upon.

File name: Supplementary Data 6.
Description: This is a summary of the included tumors along with the MAGOS clustering and event matrices from DEVOLUTION.
